# Chemical treatment rescues reduced growth of the autoimmune mutant *chs3‐2D* without compromising its immune responses

**DOI:** 10.1111/plb.70211

**Published:** 2026-03-22

**Authors:** M. Keijzer, M. Liebers, J. Seufer, L. Voll, C. Meier, S. Hoth

**Affiliations:** ^1^ Molecular Plant Physiology, Institute of Plant Science and Microbiology, Department of Biology Universität Hamburg Hamburg Germany; ^2^ Molecular Plant Physiology. Department of Biology Philipps Universität Marburg Marburg Germany; ^3^ Institute of Organic Chemistry, Department of Chemistry Universität Hamburg Hamburg Germany; ^4^ Present address: Institute of Botany Leibniz Universität Hannover Hannover Germany

**Keywords:** Autoimmunity, chemical modification, *chs3‐2D*, growth–defence trade‐off, Ro 8‐4304, salicylic acid

## Abstract

- Plant development in many species including Arabidopsis relies on the accurate balance between growth and defence. A high degree of plant immunity is often accompanied by reduced growth. On the one hand, this growth–defence trade‐off depends on the redistribution of metabolic power to either growth or defence. On the other hand, its regulation requires distinct molecular signalling pathways. Reduced growth of the Arabidopsis autoimmune mutant *chs3‐2D*, which shows strong resistance to pathogens, has been rescued by the addition of the low molecular weight compound Ro 8‐4304. The application of Ro 8‐4304, however, also resulted in a decrease of immune responses to wild‐type levels.
- We applied chemical modification of Ro 8‐4304 to identify a derivative that rescues growth of the autoimmune mutant *chs3‐2D* without compromising it immune capacity.
- In a screening of Ro 8‐4304 derivatives, removal of a fluorine atom resulted in the derivative Ro‐A03 that rescued growth, but did not affect the increased immune gene expression in *chs3‐2D*. In the presence of Ro‐A03 *chs3‐2D* seedlings showed wild‐type‐like growth, but were more resistant to bacterial pathogens than wild‐type seedlings, in line with salicylic acid levels that were higher than in the absence of Ro‐A03.
- We generated a compound by chemical modification that can efficiently rescue growth without decreasing the immune response of the *chs3‐2D* autoimmune mutant.

## Graphical abstract

We generated a compound by chemical modification that can efficiently rescue growth without decreasing the immune response of the *chs3‐2D* autoimmune mutants, thus chemically uncoupling its growth–defence trade‐off.

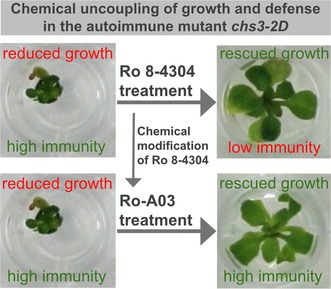

## INTRODUCTION

A central question during the life cycle of many plants is whether to grow or to defend against pathogens. Effective defence against pathogen infection is often associated with reduced growth in plants (Heil & Baldwin 2002; Tian *et al*. 2003; Kempel *et al*. 2011; Lozano‐Duran & Zipfel 2015). This apparent disadvantage of defence may be a reason for the high variability of immunity in plants (Todesco *et al*. 2010). However, plants are able to regulate the trade‐off between both growth and defence to define the optimal balance for their fitness and reproduction. This balance is also an important topic in plant breeding and thus agriculture, because breeding for maximal growth may compromise defence, whereas breeding for optimal defence may limit growth. As in both cases, yield would be decreased, it is important to find and understand mechanisms for the regulation of growth–defence trade‐offs in plants.

Plant defence relies on the recognition of pathogen infection on two different levels. At the plasma membrane, pattern recognition receptors (PRRs) recognize microbe‐associated molecular patterns (MAMPs) such as flagellin or chitin to initiate pattern‐triggered immunity (PTI). Signalling cascades are activated in PTI and induce various immune responses including an oxidative burst, changes in defence gene expression, production of antimicrobial phytoalexins, and callose appositions on the cell wall (Jones & Dangl 2006; Boller & Felix 2009; Monaghan & Zipfel 2012). Successful pathogens must suppress PTI and have evolved secretion systems to transfer effectors into the host cell to inhibit PTI (Galan *et al*. 2014; Macho & Zipfel 2015). The presence or the inhibitory function of effectors is recognized within the host cell by nucleotide‐binding leucine‐rich repeat receptors (NLRs). This recognition leads to the activation of effector‐triggered immunity (ETI), inducing the production of reactive oxygen species (ROS) and salicylic acid (SA) accumulation that eventually leads to a hypersensitve response (HR) and thus to cell death (Jones & Dangl 2006; Cui *et al*. 2015; Li *et al*. 2015; Sun *et al*. 2020). Activation of both PTI and ETI can thus reduce growth and eventually result in yield losses.

Considering a high demand of immune responses for energy, the growth–defence trade‐off may arise from the competition for resources, with reallocation from growth to immunity upon pathogen infection being a possible mechanism (Coley *et al*. 1985; Simms & Triplett 1994; Purrington 2000; Brown 2002). Indeed, the availability of carbon has been related to the susceptibility of plants to pathogens (Biemelt & Sonnewald 2006; Engelsdorf *et al*. 2013). This relationship between susceptibility and resource availability is influenced by environmental dynamics, including the availability of light in shaded locations or temperature fluctuations (de Vries *et al*. 2017). Although the competition for resources is important for growth–defence trade‐off, it has become clear in recent years that the decision to grow or to defend requires the crosstalk between growth and immune signalling, and that this decision for prioritization likely precedes the reallocation of nutrients. General molecular mechanisms are involved in the regulation of crosstalk between growth and immunity, such as chloroplast function, alternative splicing of NLR genes, miRNA‐dependent regulation, and hormone function (Huot *et al*. 2014; Eichmann & Schäfer 2015; Wasternack 2017; Figueroa‐Macias *et al*. 2021; Qi *et al*. 2024; Shen *et al*. 2024; Sun *et al*. 2024). Brassinosteroids (BRs) are hormones that regulate plant growth and development, but also influence immune responses in PTI (Albrecht *et al*. 2012; Belkhadir *et al*. 2012). The reception of BRs by its receptor kinase BRASSINOSTEROID INSENSITIVE 1 (BRI1) and of MAMPs by PRRs at the plasma membrane requires similar signalling components, including the co‐receptor BRI‐Associated Kinase 1 (BAK1) (Li *et al*. 2002; Nam & Li 2002; Chinchilla *et al*. 2007; Heese *et al*. 2007). Genetic analysis of Arabidopsis mutants lacking the expression of five repressors of jasmonic acid (JA) signalling and the light signalling receptor phyB suggested that growth and defence can be uncoupled, initiated by transcriptional rather than metabolic reprogramming (Campos *et al*. 2016). A frameshift mutation in one of the repressors, JAZ10, was sufficient to possibly uncouple the growth–defence trade‐off (Li *et al*. 2024). Upon pathogen infection, plants increase their SA content and SA‐dependent gene expression, resulting in increased defence and reduced growth (Pluharova *et al*. 2019; Peng *et al*. 2021). SA signalling requires complexes between the lipase‐like proteins ENHANCED DISEASE SUSCEPTIBILITY 1 (EDS1), PHYTOALEXIN‐DEFICIENT 4 (PAD4), and SENESCENCE‐ASSOCIATED GENE 101 (SAG101) to transduce the signal into the nucleus (Wagner *et al*. 2013; Gantner *et al*. 2019; Lapin *et al*. 2019; Sun *et al*. 2021). Recently, it has been suggested that the interaction between EDS1 and DELLA proteins, which are repressors of GA signalling (Harberd 2003), contributes to the regulation of growth–defence trade‐off *via* feedback regulation (Li *et al*. 2019).

The use of suitable systems to unravel molecular mechanisms that regulate growth–defence trade‐offs, such as the activation of ETI, are very promising. The strong activation of ETI responses in the absence of pathogen infection is referred to as plant autoimmunity. The accumulation and/or activation of NLRs in several autoimmune mutants that have a compromised homeostasis of PTI regulators, often lacking their expression due to mutation, led to reduced growth and increased defence. These responses can be induced by shifting autoimmune mutants to lower temperatures that lead to the activation of NLR signalling (Jacob *et al*. 2013; Li *et al*. 2015). The combination of inducibility and easy detection of phenotypic changes renders plant autoimmunity a very good system to apply chemical genetic screening for compounds that are able to uncouple growth and defence. Generally, chemical screening has identified several compounds with bioactivity related to plant immunity (Schreiber *et al*. 2008; Noutoshi *et al*. 2012; Joglekar *et al*. 2018; Halder *et al*. 2019). Huang *et al*. (2016) used *chilling sensitive 3*, *2D* (*chs3‐2D*) autoimmunity mutants in chemical genetic screenings. *CHS3* encodes an atypical Toll/Interleukin 1 Receptor (TIR)‐type NLR protein that regulates autoimmunity *via* the SAG101 and EDS1 pathway (Yang *et al*. 2010; Bi *et al*. 2011). In *chs3‐2D* plants, a gain‐of‐function mutation leads to the activation of constitutive defence responses (Bi *et al*. 2011). The chemical genetic screening on *chs3‐2D* seedlings identified the compound Ro 8‐4304, which suppressed autoimmune phenotypes. In the presence of Ro 8‐4304, however, low temperature‐dependent growth arrest and immune marker gene induction were both inhibited. Thus, Ro 8‐4304 did not uncouple the growth–defence trade‐off in autoimmunity of *chs3‐2D* (Huang *et al*. 2016). Interestingly, the activity of Ro 8‐4304 was dependent on the methylosome, a large protein complex that possesses an arginine methyltransferase activity. Target proteins of the methylosome are directed to the small nuclear ribonucleoprotein (snRNP) complex, which is involved in mRNA splicing (Meister & Fischer 2002). Mutations of the methylosome genes *ICln* or *SmDb3* in *chs3‐2D icln‐2* and *chs3‐2D smdb3* mutants, respectively, resulted in a decreased Ro 8‐4304 activity when compared with the Ro 8‐4304 activity in *chs3‐2D* mutants (Huang *et al*. 2016).

Here, we aimed to generate Ro 8‐4304 derivatives by chemical modification that inhibit growth arrest without affecting the strong activation of defence responses in the autoimmune mutant *chs3‐2D*. We found that the derivative Ro‐A03 rescued the reduced growth phenotype while preserving increased SA levels and the increased expression of immune genes in *chs3‐2D* mutants. In the presence of Ro‐A03, *chs3‐2D* plants showed wild‐type‐like growth but enhanced resistance to bacterial pathogens, suggesting that growth and defence responses were indeed uncoupled.

## MATERIALS AND METHODS

### Plant material and growth conditions

Seeds of Col‐0 and *chs3‐2D* were surface‐sterilized by using chlorine gas and sown in 96‐well plates on ½ MS‐medium containing 0.245% Murashige and Skoog (Duchefa, Haarlem, The Netherlands), 0.5% sucrose (Roth, Karlsruhe, Germany), and 0.8% phytoagar (Duchefa). Stock solutions were made by dissolving the compound in either DMSO (VWR International GmbH, Darmstadt, Germany), or water. The compounds dissolved in water were filter sterilized before use. Each well in the 96‐wells plate contained 3 μl from a stock solution and 147 μl ½ MS‐medium. Seeds were stratified at 4 °C for 48 h in the dark. Hereafter, they were grown under long‐day conditions (16 h of light, 8 h darkness) at the indicated temperatures, including 18 °C, 20 °C, 22 °C, and 24 °C. Plants were analysed after 3 weeks.

### Synthesis of compounds

All commercially available chemicals and reagents were used without any purification. Solvents were distilled before use, in contrast to dry solvents bought from Acros Organics (Geel, Belgium). The synthesis of Ro 8‐4304 and its analogues is described in the Data S1.

### Recording of spectra

The 1H‐, 13C‐, 19F‐, and 2D‐NMR spectra were recorded on the following Bruker (Billerica, Massachusetts, United States of America) instruments: Fourier‐300 (300 MHz/75 MHz), AV‐400 (400 MHz/100 MHz), DRX‐500 (500 MHz/125 MHz), and AVIII6000 (600 MHz/150 MHz). Chemical shifts were referenced to the solvent: Chloroform‐d (δ = 7.26 ppm in 1H‐NMR, δ = 77.16 ppm in 13C‐NMR), Methanol‐d4 (δ = 3.31 ppm and 4.87 ppm in 1H‐NMR, δ = 79.00 ppm in 13C‐NMR), DMSO‐d6 (δ = 2.50 ppm and 3.33 ppm in 1H‐NMR, δ = 39.52 ppm in 13C‐NMR), D_2_O (δ = 4.79 ppm in 1H‐NMR). Data were analysed with the MestReNova software (Mestrelab Research, Santiago de Compostela, Spain), and multiplicities are abbreviated as: s = singlet, d = doublet, dd = doublet of doublets, ddd = doublet of doublet of doublets, td = triplet of doublets, dq = doublet of quartets, t = triplet, tt = triplet of triplets, quint = quintet and m = multiplet.

ESI‐MS spectra were recorded on an Agilent 6224 ESI‐TOF mass spectrometer attached to an Agilent HPLC 1200 Series (Agilent Technologies, Santa Clara, California, USA). The EI‐MS spectra were recorded on a Thermo ISQ LT EI attached to a Thermo Trace 1300 (Thermo Fisher Scientific, Waltham, Massachusetts, USA). The data were processed using MestReNova (Mestrelab Research).

### Gene expression analysis

Plant material for quantitative PCR (qPCR) was collected after 21 days. The samples were immediately frozen in liquid nitrogen and stored at −80 °C until use. RNA was isolated using the innuPREP Plant RNA kit (Analytik Jena AG, Jena, Germany). The RNA was transformed to cDNA using the Qiagen QuantiTect^®^ reverse transcription kit (Qiagen, Hilden, Germany). For analysis of immune gene expression, qPCR was performed on RT^2^ SYBR^®^ Green qPCR master mix (Qiagen) treated samples using a Rotor‐Gene^®^ Q (Qiagen). Gene specific primers (Table S1) were synthesized by Eurofins (Hamburg, Germany). The qPCR data were analysed using qbase^+^ software, version 3.0 (Biogazelle, Zwijnaarde, Belgium) (www.qbaseplus.com). All samples were standardized to transcription levels of the reference genes *GAPDH* (AT1G13440) and *EIFα* (AT5G60390). Student's *t*‐test analysis was used to determine the significance of different values.

### Determination of salicylic acid (SA) levels

For the determination of SA levels, plants were grown for 3 days or 3 weeks on ½ MS‐medium in long‐day conditions at 18 °C in the presence of DMSO, Ro 8‐4304, and Ro‐A03, respectively. At the respective days of harvesting, 12 seedlings were pooled for each sample and stored at −80 °C after shock‐freezing in liquid nitrogen. Free SA and SAG were extracted as described previously (Sangster *et al*. 2007). HPLC separation of SA and the internal standard o‐anisic acid was performed with a binary gradient on an Agilent 1260 Infinity II system (Agilent Technologies, Inc.) equipped with a SecurityGuard Ultra guard Column (3 mm; Phenomenex) followed by an Agilent Poroshell 120 EC‐C18, 150 × 3 mm, 2.7 μm (Agilent Technologies, Inc.) at 40 °C. SAG samples were diluted 1:10 in the starting mobile phase prior to analysis. Elution began with 10% eluent A (25 mM KH_2_PO_4_, pH 2.6) and 90% eluent B (99:1 Acetonitrile:H_2_O) at a flow rate of 1 mL min^−1^, and the following gradient was applied: 0–1 min – 10% eluent B; 1–6 min – 10%–25% B; 6–9 min – 25%–50% B; 9–9.5 min – 50%–80% B; 9.5–11.5 min – 80% B; 11.5–12 min – 80%–10% B; 12–15 min – 10% B. The fluorescence detector was programmed to measure o‐anisic acid (excitation 305 nm, emission 365 nm, retention time: 5.3 min) and SA (excitation 305 nm, emission 407 nm, retention time 6.4 min). Peak identification and quantification were performed by comparison to authentic standards in the range of 1.25 to 125 ng.

### Bacterial infection assays

*Arabidopsis thaliana* Col‐0 and *chs3‐2D* plants were grown for 14 days on ½ MS‐medium at 20 °C and long‐day conditions. The growth medium contained 50 μM DMSO, Ro 8‐4304, or Ro‐A03, respectively. Plants were vacuum infiltrated with the fluorescent strain PstDC3000‐GFP of *Pseudomonas syringae* pv *tomato* at OD600 = 0.002 (Oh *et al*. 2010), and leaf discs were collected 0 and 3 days after infection. These discs were ground in a 1.5 ml tube containing 10 mM MgCl_2_ (Roth). The bacterial solutions were serial diluted and plated on NYG‐agar plates containing tetracycline (Molekula GmbH, München, Germany). They were incubated at 28 °C for 48 h. Hereafter, colony‐forming units were counted and titres were calculated.

## RESULTS

### Suppression of autoimmunity by Ro 8‐4304 is temperature‐independent

**Fig. 1:**
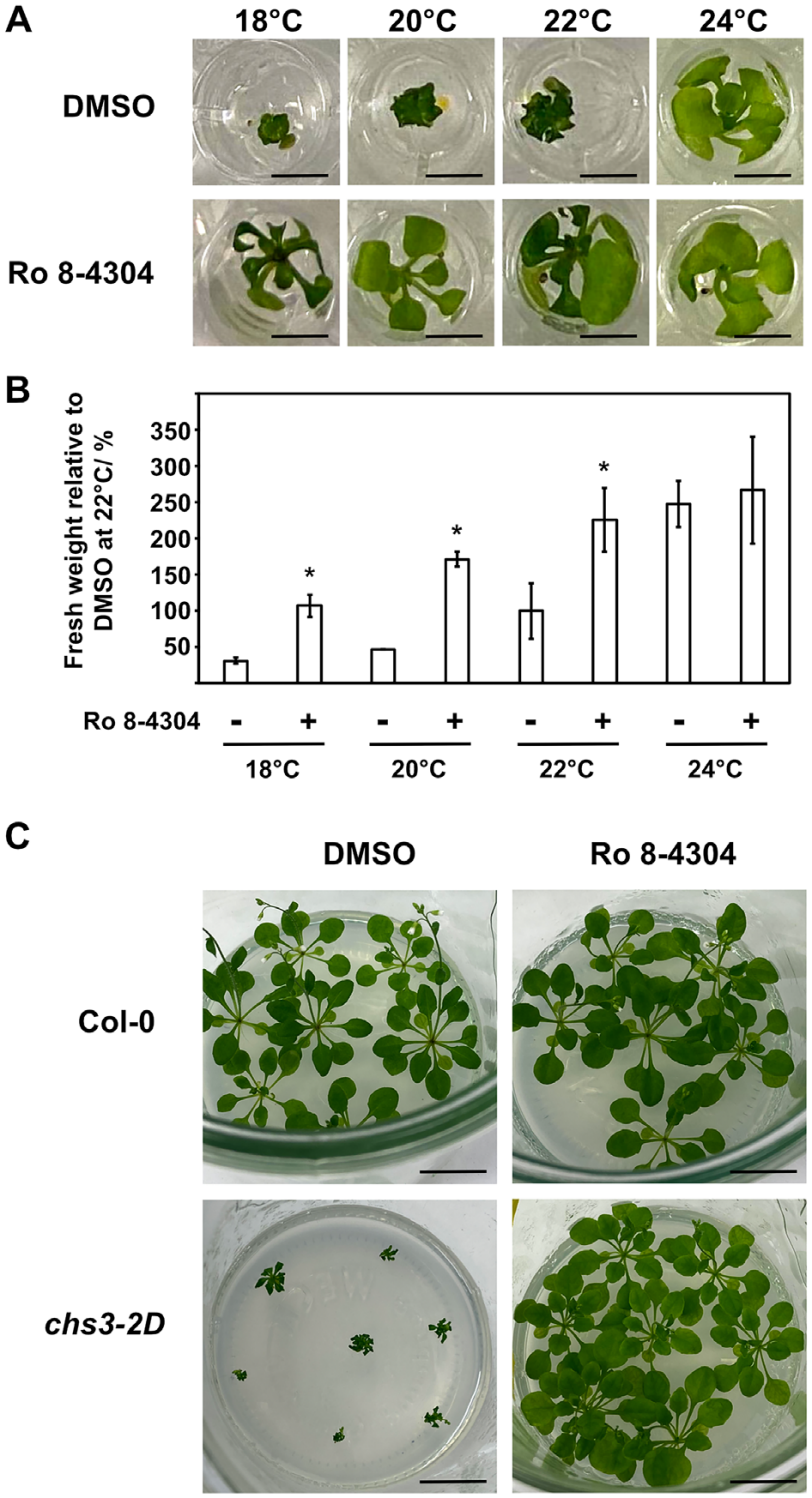
Temperature dependence of the Ro 8‐4304 effect on *chs3‐2D* growth. (A) Morphology of *chs3‐2D* plants grown in *in vitro* cultures for 21 days on solid MS media containing 15 μM Ro 8‐4304 or DMSO for control, at the indicated temperatures. The scale bars represent 0.5 cm. (B) The measured fresh weight of *chs3‐2D* plotted against the mean value of *chs3‐2D* grown on DMSO at 22 °C. The data shows the means of three replicates, and error bars represent mean ± SD (n = 3, with 10 ± 2 plants each). An asterisk indicates a significant difference to the respective DMSO control (Student's *t*‐test, *P* < 0.05). (C) Morphology of wild‐type Col‐0 and *chs3‐2D* mutant plants grown for 3 weeks on solid medium in a sterile preserving jar at 22 °C in the absence and presence of Ro 8‐4304. The scale bars represent 2 cm.

The small molecule Ro 8‐4304 was able to suppress autoimmune responses, namely reduced growth and induced immune gene expression of *chs3‐2D* mutants grown at low temperature (Huang *et al*. 2016). We examined this effect for different temperatures and showed that this suppression, which can rescue growth of *chs3‐2D* to wild‐type levels, was independent of the applied temperature (Fig. 1). Ro 8‐4304 rescued the reduced growth of *chs3‐2D* at 18 °C, 20 °C, and 22 °C, with plants generally growing larger at higher temperatures. The observed morphology and the determined relative fresh weight indicated that Ro 8‐4304 was active in this temperature range (Fig. 1A,B). Note that *chs3‐2D* did not show growth arrest at 24 °C. Additionally, we tested whether the effect of Ro 8‐4304 was stable across the development of *chs3‐2D* plants. While growth of *chs3‐2D* mutants was greatly reduced in the absence of Ro 8‐4304, plant development was fully recovered in the presence of Ro 8‐4304 when grown at 22 °C for 3 weeks (Fig. 1C). Growth and development of wild‐type Col‐0 plants were not affected by Ro 8‐4304 treatment over the long term, indicating that Ro 8‐4304 is not a general growth promoting molecule (Fig. 1C and Huang *et al*. 2016).

### Synthesis of Ro 8‐4304 derivatives by chemical modification

**Fig. 2:**
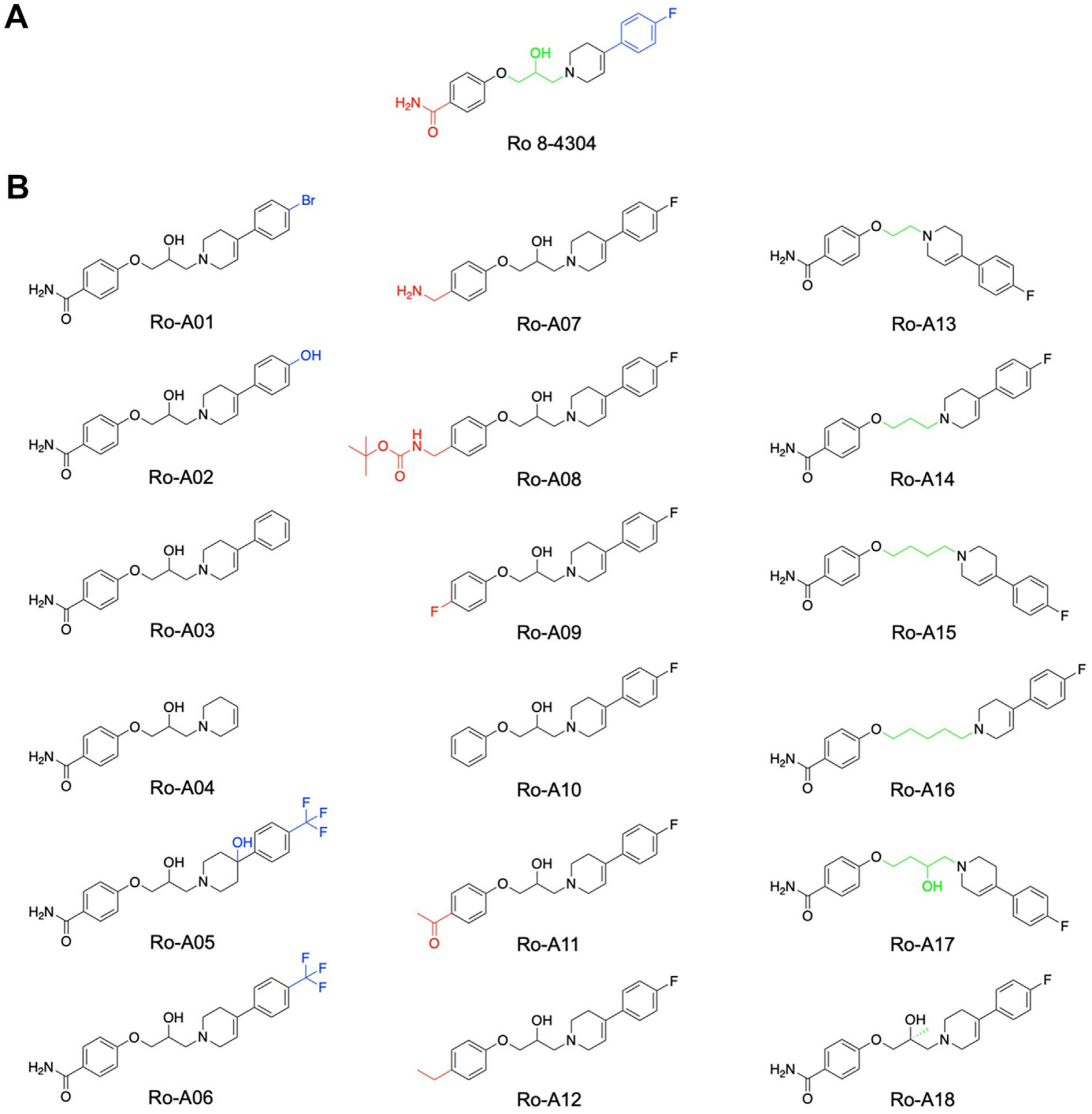
Chemical structure of Ro 8‐4304 (A) and the synthesized Ro 8‐4304 derivatives (B). The name for each derivative is given below the respective structure. The chemical change in comparison to Ro 8‐4304 is indicated by the coloured parts.

To identify derivatives of the small molecule Ro 8‐4304 that restore the reduced growth of *chs3‐2D* but do not suppress the expression of immune genes, we performed chemical modification of Ro 8‐4304 to alter different parts of the molecule. As a first step, we established the synthesis of Ro 8‐4304 following the protocol reported in the United States Patent no. 3674799A (Edenhofer & Spiegelberg, 1972). 4‐Hydroxybenzamide and epichlorohydrine were reacted overnight at room temperature (RT) in a NaOH solution to prepare 4‐oxiranylmethoxybenzamide. The product was reacted with 4‐(4‐fluorophenyl)‐1,2,3,6‐ tetrahydropyridine·HCl (PTP‐F) in EtOH, pre‐treated with triethylamine. This mixture was heated to reflux for 4 h, and Ro 8‐4304 precipitated upon cooling (Fig. 2A and Fig. S1). The Edenhofer reaction was used to synthesize various analogues by exchanging the starting materials. For example, replacement of PTP‐F by 4‐(4‐bromophenyl)‐1,2,3,6‐tetrahydropyridine·HCl gave the derivative Ro‐A01. By various changes, we were able to synthesize 14 different Ro 8‐4304 derivatives (Table S2 and Fig. 2B). A different synthetic route was used to prepare analogues Ro‐A13 to Ro‐A16 (Fig. S1). This route was adapted from Kubota *et al*. (2003) and the patent WO 2008096093A1 of (Ray *et al*. 2008). In this synthetic route, 4‐hydroxybenzamide, and for example, 1,3‐dibromopropane, were combined in ACN and K_2_CO_3_ was added. This was refluxed overnight to synthesize 4‐(4‐bromopropoxy)benzamide. After purification, this product was reacted with PTP‐F, similar to the reaction used to synthesize Ro 8‐4034. By changing the length of the alkane chain, for example, in 1,3‐dibromopropane, we were able to synthesize 4 derivatives with different chain lengths (Table S2 and Fig. 2B). The differences in structural elements or functional groups of Ro 8‐4304 in the 18 derivatives Ro‐A01 to Ro‐A18 could be used to identify parts that might be important for the activity of Ro 8‐4304.

### Compound Ro‐A03 rescues growth while immune gene induction and salicylic acid levels remain high

**Fig. 3:**
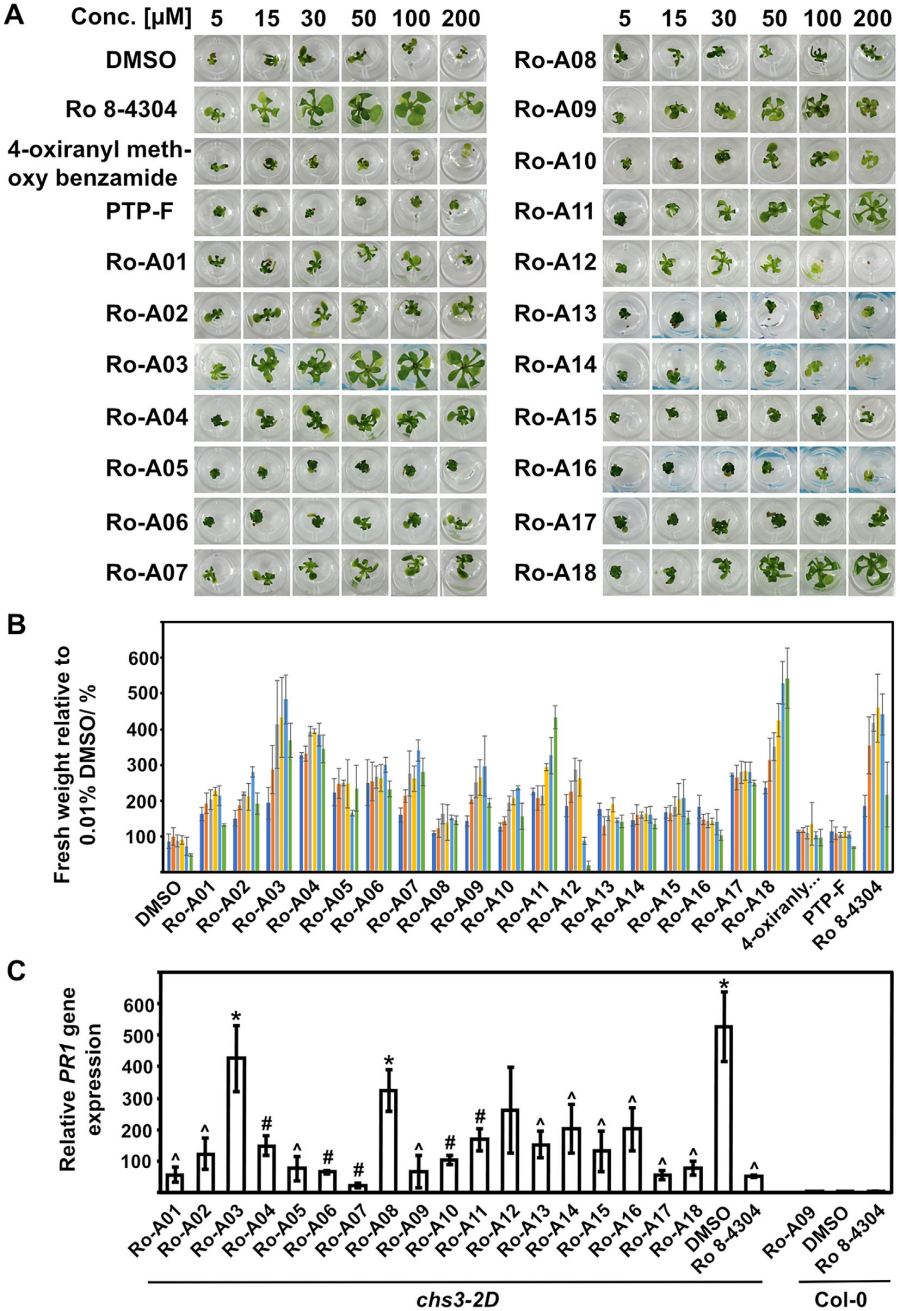
Activity of the Ro 8‐4304 derivatives on growth and immune response in *chs3‐2D* seedlings. Plants were grown for 21 ± 1 days at 18 °C and at concentrations of 5 μM (dark blue bars in (B)), 15 μM (red), 30 μM (grey), 50 μM (yellow), 100 μM (light blue), and 200 μM (green) of the respective derivative. (A) Morphology of *chs3‐2D* seedlings after treatment with different concentrations of the Ro 8‐4304 derivatives. (B) Fresh weight of *chs3‐2D* relative to the mean fresh weight of *chs3‐2D* grown in the presence of 0.01% DMSO. The data show the mean values of three replicates, and error bars represent mean ± SD (n = 3, with 8–12 plants each). Statistical significances of the differences are given in Fig. S2. (C) Relative expression of the *PR1* gene in *chs3‐2D* seedlings grown at 18 °C for 21 days and treated with the respective derivative at a concentration of 15 μM. Mean values of three replicates each are given, and error bars represent the standard deviation. The carets indicate a significant difference of *P* < 0.05 in comparison to the values of *chs3‐2D* treated with 0.01% DMSO. The asterisks indicate a significant difference of *P* < 0.05 in comparison to the values of *chs3‐2D* treated with 15 μM Ro 8‐4304. The hashtags indicate a significant difference of *P* < 0.05 in comparison to both, to the values of *chs3‐2D* treated with 0.01% DMSO and to the values of *chs3‐2D* treated with 15 μM Ro 8‐4304. The significances were calculated using the Student's *t*‐test.

The 18 newly synthesized Ro 8‐4304 derivatives were used in concentrations of 5 μM, 15 μM, 30 μM, 50 μM, 100 μM, and 200 μM to treat *chs3‐2D* mutant seedlings grown at 18 °C. While in the absence of Ro 8‐4304 in the DMSO control *chs3‐2D* showed the expected reduction of growth, Ro 8‐4304 restored growth at all concentrations (Fig. 3A). Growth in the presence of the two building blocks of Ro 8‐4304, namely, 4‐oxiranylmethoxy benzamide and PTP‐F (c.f. Fig. S1), as well as in the presence of the derivatives Ro‐A05, Ro‐A06, Ro‐A08, and Ro‐A13 to Ro‐A17 did not result in morphological changes when compared with growth in the DMSO control. Slight changes in morphology were observed in the presence of Ro‐A01, Ro‐A02, Ro‐A07, Ro‐A09 to Ro‐A12. The appearance of *chs3‐2D* plants in the presence of Ro‐A03, Ro‐A04, and Ro‐A18 suggested that the development was restored to a similar degree as for Ro 8‐4304 (Fig. 3A). To quantify these morphological changes, we determined the fresh weight of *chs3‐2D* seedlings grown for 3 weeks in the presence of the respective small molecules and concentrations (Fig. 3B). While in DMSO *chs3‐2D* seedlings were strongly reduced in growth, the presence of Ro 8‐4304 was sufficient to restore growth, indicated by a significant increase in the fresh weight (Fig. 3B). A similar increase in fresh weight was also determined for *chs3‐2D* seedlings grown in the presence of Ro‐A03, Ro‐A04, Ro‐A11, and Ro‐A18 (Fig. 3B). Except for the two starting compounds for the synthesis of Ro 8‐4304, which could not change growth of *chs3‐2D*, all other Ro 8‐4304 derivatives still restored growth but less effective than Ro 8‐4304. For better resolution, the significances were given in separate graphs (Fig. S2).

**Fig. 4:**
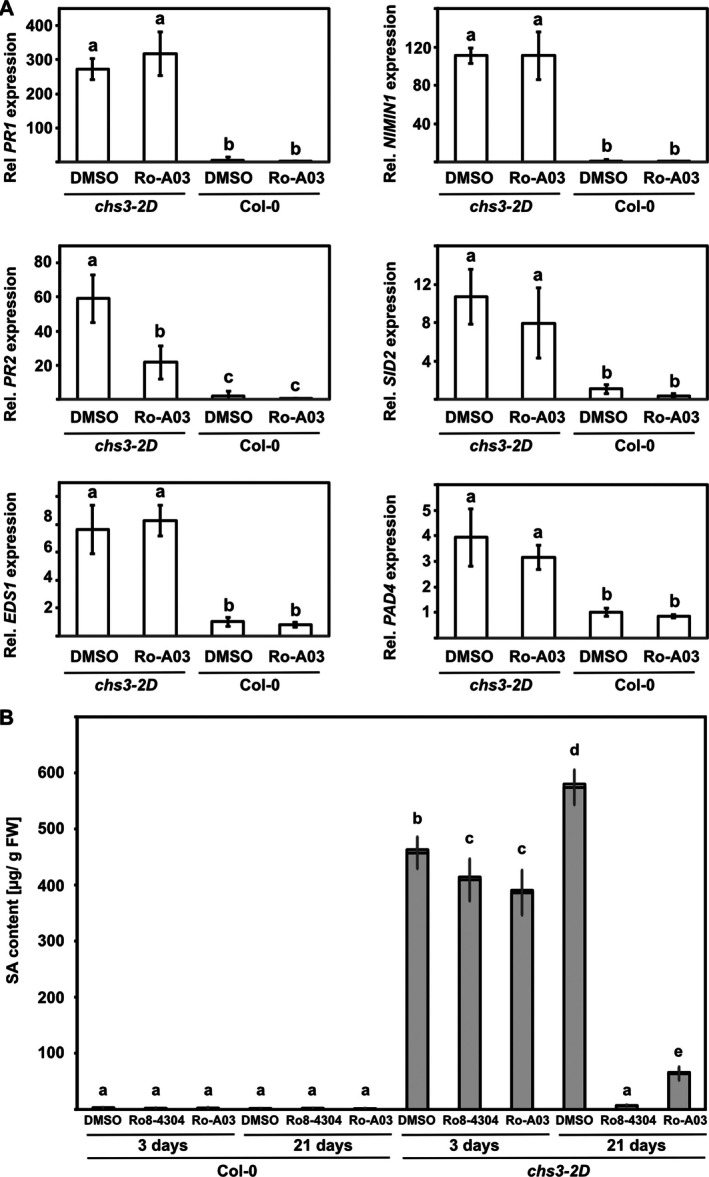
Immune responses in *chs3‐2D* seedlings in the absence or presence of 50 μM Ro‐A03. (A) Defence gene expression in *chs3‐2D* seedlings in the absence or presence of 50 μM Ro‐A03. Plants were grown at 18 °C for 21 days. The relative expression is shown for the indicated immune genes *PR1*, *NIMIN1*, *PR2*, *SID2*, *EDS1*, and *PAD4*. The data represent mean ± SD (n = 3). Different letters indicate significant differences between groups, whereas identical letters denote no significant differences (Student's *t*‐test, *P* < 0.05). (B) SA content in 3‐week‐old *chs3‐2D* and Col‐0 wild‐type seedlings grown at 18 °C in the absence or presence of 50 μM Ro‐A03 or Ro 8‐4304. Levels of free SA (white) and conjugated SA (SAG) (grey) are shown for Col‐0 and *chs3‐2D* plants that were incubated for 3 and 21 days, respectively. The data represent the mean ± SE of five biological replicates with 12 plants each. Different letters on top of the bars indicate significant differences, whereas identical letters denote no significant differences (two‐way Anova, LSD test, *P* < 0.05).

In addition to the described morphological changes, we analysed the expression of the immune genes *PR1* and *NIMIN1* in *chs3‐2D* seedlings grown in the presence of the respective synthesized compounds to investigate immune responses on the molecular level (Fig. 3C and Fig. S3). No significant expression of both genes was observed in wild‐type plants. As expected, the expression of both *PR1* and *NIMIN1* was on a high level in *chs3‐2D* seedlings grown in the DMSO control, because autoimmunity was induced at 18 °C. The presence of Ro 8‐4304 in the growth medium reduced the expression of *PR1* to almost wild‐type levels and significantly diminished the expression of *NIMIN1*. While most Ro 8‐4304 derivatives that restored growth of *chs3‐2D* only partially also mediated reduced expression of immune genes, the derivative Ro‐A03 was an exception. Ro‐A03 restored growth of *chs3‐2D* to comparable levels as Ro 8‐4304 did (Fig. 3B). However, in the presence of Ro‐A03 the expression of *PR1* and *NIMIN1* was similar to the expression in the DMSO control (Fig. 3C and Fig. S3). In a next step, we tested whether a higher concentration of Ro‐A03 of 50 μM, which restored growth more efficiently than a concentration of 15 μM (Fig. 3B), resulted in identical expression of immune genes. Indeed, the expression of all tested genes *PR1*, *NIMIN1*, *SID2*, *EDS1*, and *PAD4* was identical in *chs3‐2D* seedlings grown in the presence of DMSO or Ro‐A03, respectively (Fig. 4A). Only the expression of *PR2* was slightly lower in the presence of Ro‐A03. As the induction of the studied immune genes is triggered by SA signalling, the SA pool was examined in wild‐type and *chs3‐2D* plants grown in the absence and in the presence of Ro 8‐4304 or Ro‐A03, respectively. Whereas in all conditions SA levels stayed low in Col‐0 wild‐type plants, free and conjugated SA increased in *chs3‐2D* seedlings (Fig. 4B). Incubation for 3 days on Ro 8‐4304 or Ro‐A03 resulted in a slight decrease of SA when compared with the DMSO control. Whereas the SA content was further increased in *chs3‐2D* plants grown for 21 days on DMSO, the SA content in *chs3‐2D* plants was fully reduced back to wild‐type levels after 21 days of growth in the presence of Ro 8‐4304. In *chs3‐2D* plants grown for 21 days in presence of Ro‐A03, the SA content was also reduced with respect to the DMSO controls. However, the SA content stayed on a level that was about 45‐fold and 10‐fold higher when compared with the levels in wild‐type plants or in *chs3‐2D* plants grown for 21 days in the presence of Ro 8‐4304, respectively (Fig. 4B).

Apparently, the restoration of growth by Ro‐A03 was uncoupled from immune gene induction and SA content in *chs3‐2D* seedlings, thus suggesting that *chs3‐2D* plants with restored growth may be more resistant to pathogens.

### Compound Ro‐A03 conferred higher pathogen resistance to *chs3‐2D* plants with restored growth

**Fig. 5:**
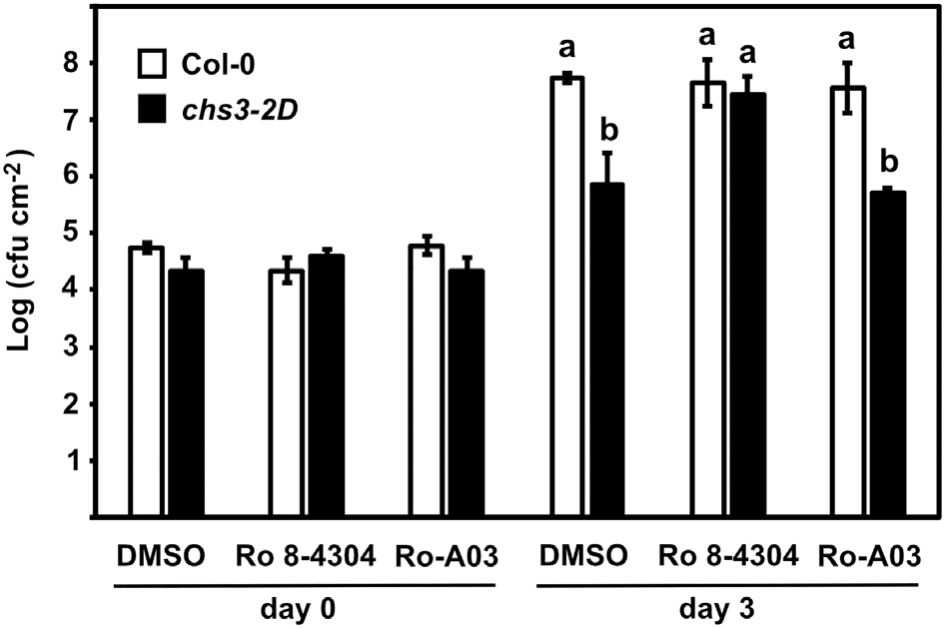
The effect of Ro‐A03 on resistance of *chs3‐2D* to the bacterial pathogen *P.s.t*. DC3000. Col‐0 and *chs3‐2D* plants were infected with the *Pseudomonas syringae* pv tomato strain *P.s.t*. DC3000‐GFP in the presence of DMSO, 50 μM Ro 8‐4304, or 50 μM Ro‐A03. Plants were grown for 2 weeks at 20 °C prior to vacuum infiltration with *P.s.t*. DC3000‐GFP. Quantification of colony‐forming units (CFU) at 0 and 3 days post‐inoculation. Data represent means of three replicates with four plant discs per replicate. Error bars are ± SD. Different letters indicate significant differences between groups, whereas identical letters denote no significant differences (Student's *t*‐test, *P* < 0.05).

The maintained induction of immune gene expression in *chs3‐2D*, in which growth was restored by Ro‐A03, prompted us to test for pathogen resistance of *chs3‐2D* in the presence of Ro‐A03. For that purpose, we grew *chs3‐2D* seedlings for 14 days at 20 °C in the presence of 50 μM Ro‐A03, of Ro 8‐4304 as positive control, and of DMSO as negative control. At day 14, seedlings were infected with the *Pseudomonas syringae* pv tomato strain *P.s.t*. DC3000‐GFP (Oh *et al*. 2010). In the presence of Ro 8‐4304 plant growth was restored in *chs3‐2D* (Fig. 1). However, the reduction of immune gene expression to wild‐type levels by Ro 8‐4304 that was previously published and confirmed for additional immune genes (c.f. Figs 3 and 4; Fig. S3; Huang *et al*. 2016) resulted in enhanced growth of *P.s.t*. DC3000‐GFP and thus in decreased resistance in *chs3‐2D* seedlings (Fig. 5). In contrast to Ro 8‐4304, its derivative Ro‐A03 restored growth but allowed for higher expression of immune genes (Figs 3 and 4; Fig. S3). In line with that bacterial growth in *chs3‐2D* seedlings grown in the presence of Ro‐A03 was reduced, indicating resistance to *P.s.t*. DC3000‐GFP (Fig. 5). While bacterial growth in *chs3‐2D* in the presence of Ro 8‐4304 was as high as in wild‐type plants, the addition of Ro‐A03 significantly reduced bacterial growth (Fig. 5), indicating that immune responses stayed high although growth was restored to wild‐type‐like growth.

### The dependence of Ro 8‐4304 activity on the methylosome was marginally changed by the chemical modification in Ro‐A03

**Fig. 6:**
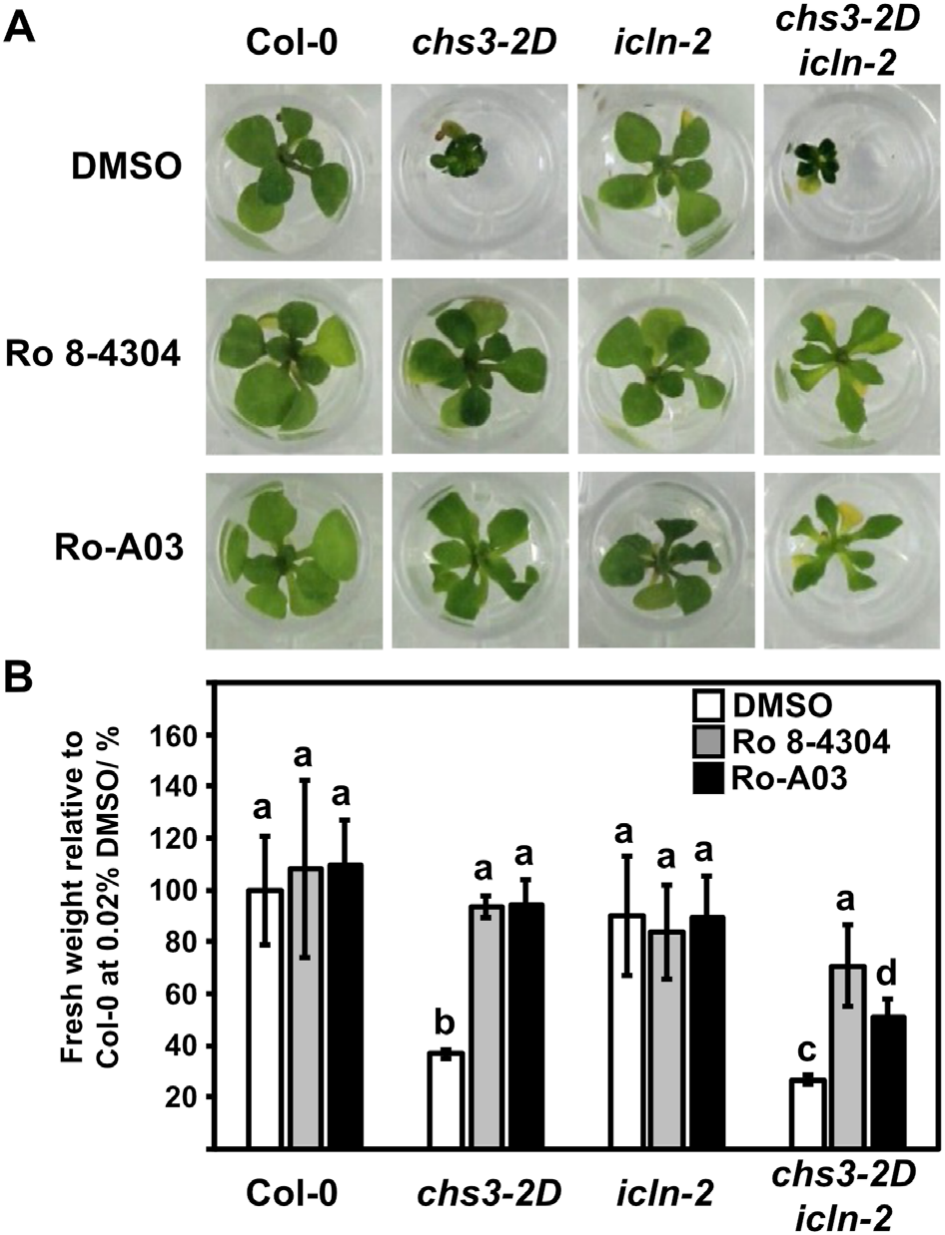
The impact of Ro‐A03 on the methylosome mutant *icln‐2*. (A) Morphology of Col‐0, *chs3‐2D*, *icln‐2*, and *chs3‐2D icln‐2* seedlings grown for 21 days at 18 °C on DMSO control or in the presence of Ro 8‐4304 and Ro‐A03, respectively, in a concentration of 50 μM. (B) The measured fresh weight of the mutants plotted relative to the mean value of Col‐0 grown on 0.02% DMSO. The data show the means of three replicates, and error bars represent ± SD (n = 3, with 8–12 plants each). Different letters indicate significant differences between groups, whereas identical letters denote no significant differences (Student's *t*‐test, *P* < 0.05).

The rescue of growth in *chs3‐2D* mutants in the presence of Ro 8‐4304 required an intact methylosome. The effect was lost in *chs3‐2D smd3b* and *chs3‐2D icln‐1* double mutants that lack expression of the methylosome components SmD3b and AtICln, respectively (Huang *et al*. 2016). To investigate whether this dependence was maintained for the effect of Ro‐A03, we investigated the growth of *icln‐2* single and *chs3‐2D icln‐2* double mutants in the presence of Ro‐A03 in comparison to *chs3‐2D*. In line with previous data, growth of *icln‐2* mutants was not affected by Ro 8‐4304 (Huang *et al*. 2016), and was also not changed upon treatment with Ro‐A03 (Fig. 6A,B). The growth rescue in *chs3‐2D icln‐2* double mutants in the presence of Ro 8‐4304 and Ro‐A03 was reduced for both treatments to almost the same level (Fig. 6A,B), indicating that the dependence on the methylosome did not generally change. Due to the small but significant difference between both treatments we can, however, not exclude that the chemical modification in Ro‐A03 may slightly affect methylosome function.

## DISCUSSION

Plant growth and immunity are regulated by small organic molecules, including plant hormones. Chemical genetic screening has proven to be a powerful tool for identifying chemical compounds that regulate growth and immunity (Lepri *et al*. 2023). The inhibitor of cell expansion hypostatin, compounds for priming immune responses or altering growth, regulators of hypersensitive cell death, PTI regulators, effectors of hormone signalling, small molecules affecting strigolactone accumulation, and the seed germination inhibitor pyrabactin, which was used to identify the abscisic acid (ABA) receptor PYRABACTIN RESISTANCE 1, were identified in such screens (Serrano *et al*. 2007; Zhao *et al*. 2007; Park *et al*. 2009; Tsuchiya *et al*. 2010; Noutoshi *et al*. 2012; Noutoshi & Shirasu 2018; Chini *et al*. 2021; Emmenecker *et al*. 2025). High‐throughput screening also led to the identification of Ro 8‐4304 that has been shown to rescue reduced growth of the autoimmune mutant *chs3‐2D* to wild‐type growth and development (Huang *et al*. 2016; Fig. 1). Here, we aimed to change and eventually improve the properties of Ro 8‐4304 and synthesized 18 derivatives to study their properties. The synthesis and targeted modification of growth‐ and immunity‐affecting compounds is a promising approach in fundamental research and in agriculture (Zhou & Wang 2018). Chemical modification generated various halogenated derivatives of SA by the addition of chloro and fluoro atoms, respectively, that induced higher resistance to pathogens than SA (Conrath *et al*. 1995; Silverman *et al*. 2005). The SA derivative benzoylsalicylic acid (BzSA) was modified by chemical synthesis to produce different compounds that were slightly more active than BzSA (Kamatham *et al*. 2017). Activity of several synthesized derivatives of the defence‐regulating plant hormone jasmonic acid (JA) or of the JA mimic coronatine was also proven (Krumm *et al*. 1995; Pluskota *et al*. 2007; Monte *et al*. 2014). Synthetic JA agonists were recently shown to promote small changes in defence without reducing plant yield in rice, and may open new avenues for disease control (Xiao *et al*. 2025).

The results of chemical modification of Ro 8‐4304 shown in Figs 2 and 3; Figs S2 and S3 will be discussed in the following paragraph. Ro 8‐4304 consists of two major parts that contain ring structures and that are connected with each other *via* a linker. Both parts were important for the efficiency of Ro 8‐4304, because both building blocks 4‐oxiranylmethoxy benzamide and PTP‐F, which resemble the two major parts, failed to achieve rescue of *chs3‐2D* autoimmunity. In derivatives Ro‐A13 to Ro‐A16, we changed the length of the linker and deleted also the hydroxy group comprised in the linker. These changes resulted in the loss of activity with respect to the reduction in growth, and immune gene expression was also not significantly changed. Derivative Ro‐A17 has a slightly longer linker, but still comprised the hydroxy group. Treatment with Ro‐A17 resulted in an intermediate rescue of growth, and immune gene expression upon Ro‐A17 was comparable to Ro 8‐4304. Apparently, the hydroxy group in the linker region is important for Ro 8‐4304 activity. Introducing an additional methyl group next to the hydroxy group increased the capacity of Ro‐A18 to rescue growth and to decrease immune gene expression. The electron donating property or the steric effects of the methyl group thus did not generally change the efficiency of the hydroxy group Ro 8‐4304. Chemical modifications of the benzamide side of Ro 8‐4304 changed Ro 8‐4304 properties differently. Removing the oxygen from the benzamide (Ro‐A07) and exchanging the acid amide by fluorine (Ro‐A09) or an ethyl group (Ro‐A12), respectively, rescued growth of *chs3‐2D*, but less effective than Ro 8‐4304, although the expression of immune genes appeared slightly reduced compared with Ro 8‐4304. Changing the amine for a methyl group (Ro‐A11) led to almost the same degree of growth rescue. Taken together, chemical modifications of the benzamide side did not strongly affect the properties of Ro 8‐4304. Only when introducing a chemical group for the acid amide that was much larger (Ro‐A08), the reduced growth could not be rescued. Correspondingly, the expression of *PR1* was still high upon Ro‐A08 treatment. Finally, we introduced changes to the fluorine atom in Ro 8‐4304. This electron withdrawing atom was changed for bromine (Ro‐A01), which has a weaker electron withdrawing effect, but a higher atomic radius. Nonetheless, this modification was not very effective. Changing the fluorine to an electron donating alcohol group (Ro‐A02) also resulted in properties comparable to Ro‐A01, indicating that the electronic properties were not important for Ro 8‐4304 activity. Introducing larger groups in Ro‐A05 and Ro‐A06 did also not strongly change the properties of Ro 8‐4304, except for a less pronounced growth rescue, suggesting that the increased size prevents these compounds from binding to a putative binding protein. Reduced growth, however, was rescued as efficiently as in Ro 8‐4304 when removing the fluorine (Ro‐A03) or even a larger part (Ro‐A04), showing that this side is less important for the impact of Ro 8‐4304 on growth. Interestingly, although still rescuing growth, Ro‐A03 did not reduce immune gene expression (Figs. 3 and 4; Fig. S2), indicating that growth and immunity were not coupled anymore. In line with that defence of *chs3‐2D* was as effective in the presence of Ro‐A03 as in the presence of DMSO only (Fig. 5). Taken together, chemical modification of Ro 8‐4304 generated changes in its properties, in particular in the derivative Ro‐A03 lacking the fluorine atom. A similar effect on activity was seen in SA signalling, where the addition of a fluorine atom to SA increased its potential for plant defence (Silverman *et al*. 2005).

The mechanism for recognition of Ro 8‐4304 is not known, as no binding protein was found so far. It has been previously shown that activity of Ro 8‐4304 on *chs3‐2D* depends on components of the methylosome, which in eukaryotic cells plays a role in the biogenesis of small nuclear ribonucleoproteins required for spliceosome function, and splicing has been suggested to regulate growth–defence trade‐offs (Meister & Fischer 2002; Huang *et al*. 2016; Sun *et al*. 2024). We determined that Ro 8‐4304 and Ro‐A03 likely require the same molecular mechanism for the rescue of growth in *chs3‐2D* plants (Fig. 6). To unravel the signalling pathway that is used by Ro 8‐4304 and Ro‐A03, it will be important to identify a binding protein. This may also help to investigate why Ro‐A03 is able to rescue growth of *chs3‐2D* without reducing the expression of immune genes and thus resistance. We could show that in the presence of Ro 8‐4304 and Ro‐A03, respectively, growth of *chs3‐2D* plants was rescued. The accumulation of SA to 574.2 μg/gFW^−1^, which was observed in *chs3‐2D* autoimmunity under control conditions, was less pronounced already after 3 days of growth in the presence of Ro 8‐4304 or Ro‐A03 and was compromised to a large extent after 21 days of growth in the presence of either of the two chemicals (Fig. 4). This indicated that treatment with Ro 8‐4304 or Ro‐A03 generally leads to suppression of SA accumulation in the autoimmune mutant *chs3‐2D*, regardless of whether treatment is short (3 days) or long (21 days) and apparently more pronounced over the course of treatment. Interestingly, longer treatment resulted in differences in the SA content between Ro 8‐4304‐ and Ro‐A03‐treated *chs3‐2D* plants. When grown in the presence of Ro‐A03 for 21 days, the SA content of *chs3‐2D* mutants was still significantly increased (63.7 μg/gFW^−1^) compared with the SA content in *chs3‐2D* seedlings grown for 21 days in the presence of Ro 8‐4304 (6.3 μg/gFW^−1^) or in wild‐type plants (1.4 μg/gFW^−1^) (Fig. 4B). Such a moderate but significant SA accumulation suggested that Ro‐A03‐treated *chs3‐2D* plants, in contrast to Ro 8‐4304‐treated *chs3‐2D* plants, may have higher resistance to pathogen infection, because it has been shown previously that only 10 μg/g^−1^ fresh weight (gFW) of total SA or 50 μg/gFW^−1^ of SAG were necessary for an effective immune response in Arabidopsis (Wildermuth *et al*. 2001; Yang *et al*. 2010). Based on the measurements taken after 3 and 21 days of treatment, we decided to test resistance of Ro‐A03‐treated plants after 14 days of growth and exposed the plants to bacterial pathogens for 3 days in accordance with standard infection protocols. We were indeed able to show that the *chs3‐2D* plants after longer treatment with Ro‐A03 for these 17 days were more resistant to a bacterial pathogen than *chs3‐2D* plants treated with Ro 8‐4304 (Fig. 5). However, the suggested correlation between the higher SA content with higher resistance in Ro‐A03‐ *versus* Ro 8‐4304‐treated *chs3‐2D* plants has still to be proven. The exact involvement of SA‐dependent processes, which may lead to the sustained immune gene expression and resistance of Ro‐A03‐treated compared with Ro 8‐4304‐treated *chs3‐2D* plants, needs to be investigated in future studies. This could also help to clarify how the effect at the molecular level differs between Ro 8‐4304 and its derivative Ro‐A03. Taken together, Ro‐A03 mediated uncoupling may be the starting point to unravel additional regulations of growth–defence trade‐offs.

Generally, plant hormones are small organic molecules that mediate the regulation of growth and defence (Huot *et al*. 2014; Berens *et al*. 2017; Aerts *et al*. 2021). The performance of plants depends on the successful recognition of changes in the environment and the implementation into an integrated response to abiotic and biotic stimuli. This often involves the synthesis of hormones and changes in hormone signalling. As plant hormones not only regulate growth and development, but are also involved in the regulation of immune responses (Tsuda *et al*. 2009), hormone crosstalk may be central to the regulation of growth–defence trade‐off. This has been observed in several interactions between the classical defence‐related hormones JA or SA and hormones, which were described as growth‐regulating hormones, including ABA, GA, and ethylene (Pieterse *et al*. 2009; Shigenaga & Argueso 2016). For example, the MYC transcription factor MYC2 and the ERF transcription factor ORA59 are hubs in JA signalling. Both transcription factors represent important points of intersection with other hormone signalling pathways (Aerts *et al*. 2021), such as ABA signalling through the interaction of MYC2 with the ABA receptor PYL6 (Aleman *et al*. 2016). In Arabidopsis and rice the antagonism between GA and JA signalling has been shown to determine the trade‐off between growth and defence (Campos *et al*. 2016). Recently, it has been suggested that the antagonism between the kinase complexes SnRK1 and TOR may effectively regulate the balancing between growth and defence (Margalha *et al*. 2019). Generally, the regulation of growth–defence trade‐off may also involve changes in cell cycle control that is largely determined by hormone signalling networks (Eichmann & Schäfer 2015). It will be interesting to investigate the effect of Ro 8‐4304 and Ro‐A03 on the different molecular pathways that appear to decide about how to balance growth and defence for optimal plant performance and reproduction.

## AUTHOR CONTRIBUTIONS

SH designed the study. MK, CM, LV, and SH designed the experiments. MK, ML, and JS performed the experiments and data analyses. MK and SH drafted the manuscript. All authors discussed and interpreted results, revised the manuscript, and approved it.

## Supporting information


**Table S1.** Primers used for PCR experiments.
**Table S2.** Starting compounds for synthesis of Ro 8‐4304 derivatives.
**Fig. S1.** Synthesis of derivatives of Ro 8‐4304.
**Fig. S2.** The relative fresh weight of chs3‐2D treated with different concentrations of DMSO, Ro 8‐4304, 4‐oxiranyl methoxybenzamide, and PTP‐F, as well as the derivatives Ro‐A01 to Ro‐A18.
**Fig. S3.** Relative expression of the NIMIN1 gene in chs3‐2D seedlings grown at 18 °C for 21 days and treated with the respective compound at a concentration of 15 μM.

## Data Availability

All data supporting the findings of this study are available within the paper and within its Supporting Information – S1.

## References

[plb70211-bib-0001] Aerts N. , Pereira Mendes M. , van Wees S.C.M. (2021) Multiple levels of crosstalk in hormone networks regulating plant defense. Plant Journal, 105, 489–504. 10.1111/tpj.15124 PMC7898868 33617121

[plb70211-bib-0002] Albrecht C. , Boutrot F. , Segonzac C. , Schwessinger B. , Gimenez‐Ibanez S. , Chinchilla D. , Rathjen J.P. , de Vries S.C. , Zipfel C. (2012) Brassinosteroids inhibit pathogen‐associated molecular pattern‐triggered immune signaling independent of the receptor kinase BAK1. Proceedings of the National Academy of Sciences of he United States of America, 109, 303–308. 10.1073/pnas.1109921108 PMC3252947 22087006

[plb70211-bib-0003] Aleman F. , Yazaki J. , Lee M. , Takahashi Y. , Kim A.Y. , Li Z. , Kinoshita T. , Ecker J.R. , Schroeder J.I. (2016) An ABA‐increased interaction of the PYL6 ABA receptor with MYC2 transcription factor: a putative link of ABA and JA signaling. Scientific Reports, 6, 28941. 27357749 10.1038/srep28941 PMC4928087

[plb70211-bib-0004] Belkhadir Y. , Jaillais Y. , Epple P. , Balsemao‐Pires E. , Dangl J.L. , Chory J. (2012) Brassinosteroids modulate the efficiency of plant immune responses to microbe‐associated molecular patterns. Proceedings of the National Academy of Sciences, 109, 297–302. 10.1073/pnas.1112840108 PMC3252953 22087001

[plb70211-bib-0005] Berens M.L. , Berry H.M. , Mine A. , Argueso C.T. , Tsuda K. (2017) Evolution of hormone signaling networks in plant defense. Annual Review of Phytopathology, 55, 401–425. 10.1146/annurev-phyto-080516-035544 28645231

[plb70211-bib-0006] Bi D. , Johnson K.C. , Zhu Z. , Huang Y. , Chen F. , Zhang Y. , Li X. (2011) Mutations in an atypical TIR‐NB‐LRR‐LIM resistance protein confer autoimmunity. Frontiers in Plant Science, 2, 71. 22639607 10.3389/fpls.2011.00071 PMC3355616

[plb70211-bib-0007] Biemelt S. , Sonnewald U. (2006) Plant‐microbe interactions to probe regulation of plant carbon metabolism. Journal of Plant Physiology, 163, 307–318. 16368160 10.1016/j.jplph.2005.10.011

[plb70211-bib-0008] Boller T. , Felix G. (2009) A renaissance of elicitors: perception of microbe‐associated molecular patterns and danger signals by pattern‐recognition receptors. Annual Review of Plant Biology, 60, 379–406. 10.1146/annurev.arplant.57.032905.105346 19400727

[plb70211-bib-0009] Brown J.K. (2002) Yield penalties of disease resistance in crops. Current Opinion in Plant Biology, 5, 339–344. 12179968 10.1016/s1369-5266(02)00270-4

[plb70211-bib-0010] Campos M.L. , Yoshida Y. , Major I.T. , de Oliveira Ferreira D. , Weraduwage S.M. , Froehlich J.E. , Johnson B.F. , Kramer D.M. , Jander G. , Sharkey T.D. , Howe G.A. (2016) Rewiring of jasmonate and phytochrome B signalling uncouples plant growth‐defense tradeoffs. Nature Communications, 7, 12570. 10.1038/ncomms12570 PMC5155487 27573094

[plb70211-bib-0011] Chinchilla D. , Zipfel C. , Robatzek S. , Kemmerling B. , Nurnberger T. , Jones J.D. , Felix G. , Boller T. (2007) A flagellin‐induced complex of the receptor FLS2 and BAK1 initiates plant defence. Nature, 448, 497–500. 17625569 10.1038/nature05999

[plb70211-bib-0012] Chini A. , Monte I. , Fernandez‐Barbero G. , Boter M. , Hicks G. , Raikhel N. , Solano R. (2021) A small molecule antagonizes jasmonic acid perception and auxin responses in vascular and nonvascular plants. Plant Physiology, 187, 1399–1413. 34618088 10.1093/plphys/kiab369 PMC8566257

[plb70211-bib-0013] Coley P.D. , Bryant J.P. , Chapin F.S., 3rd (1985) Resource availability and plant antiherbivore defense. Science, 230, 895–899. 17739203 10.1126/science.230.4728.895

[plb70211-bib-0014] Conrath U. , Chen Z. , Ricigliano J.R. , Klessig D.F. (1995) Two inducers of plant defense responses, 2,6‐dichloroisonicotinec acid and salicylic acid, inhibit catalase activity in tobacco. Proceedings of the National Academy of Sciences, 92, 7143–7147. 10.1073/pnas.92.16.7143 PMC41295 11607566

[plb70211-bib-0015] Cui H. , Tsuda K. , Parker J.E. (2015) Effector‐triggered immunity: from pathogen perception to robust defense. Annual Review of Plant Biology, 66, 487–511. 10.1146/annurev-arplant-050213-040012 25494461

[plb70211-bib-0016] de Vries J. , Evers J.B. , Poelman E.H. (2017) Dynamic plant‐plant‐herbivore interactions govern plant growth‐defence integration. Trends in Plant Science, 22, 329–337. 28089490 10.1016/j.tplants.2016.12.006

[plb70211-bib-0017] Edenhofer A. , Spiegelberg H. (1972) 4'‐Phenyl‐3,6‐dihydro‐1‐(2H)pyridyl‐2‐hydroxypropoxy‐anilides and derivatives thereof. United States Patents, 3(674), 799A.

[plb70211-bib-0018] Eichmann R. , Schäfer P. (2015) Growth versus immunity – a redirection of the cell cycle? Current Opinion in Plant Biology, 26, 106–112. 26190589 10.1016/j.pbi.2015.06.006

[plb70211-bib-0019] Emmenecker C. , Dai J. , Lefranc S. , Ouddah A. , Guerin J. , Pakzad S. , Andrey P. , Kumar R. (2025) A high‐throughput differential chemical genetic screen uncovers genotype‐specific compounds altering plant growth. iScience, 28, 112375. 40292320 10.1016/j.isci.2025.112375 PMC12032938

[plb70211-bib-0020] Engelsdorf T. , Horst R.J. , Pröls R. , Pröschel M. , Dietz F. , Hückelhoven R. , Voll L.M. (2013) Reduced carbohydrate availability enhances the susceptibility of Arabidopsis toward colletotrichum higginsianum. Plant Physiology, 162, 225–238. 23487433 10.1104/pp.112.209676 PMC3641204

[plb70211-bib-0021] Figueroa‐Macias J.P. , Garcia Y.C. , Nunez M. , Diaz K. , Olea A.F. , Espinoza L. (2021) Plant growth‐defense trade‐offs: molecular processes leading to physiological changes. International Journal of Molecular Sciences, 22, 693. 33445665 10.3390/ijms22020693 PMC7828132

[plb70211-bib-0022] Galan J.E. , Lara‐Tejero M. , Marlovits T.C. , Wagner S. (2014) Bacterial type III secretion systems: specialized nanomachines for protein delivery into target cells. Annual Review of Microbiology, 68, 415–438. 10.1146/annurev-micro-092412-155725 PMC4388319 25002086

[plb70211-bib-0023] Gantner J. , Ordon J. , Kretschmer C. , Guerois R. , Stuttmann J. (2019) An EDS1‐SAG101 complex is essential for TNL‐mediated immunity in *Nicotiana benthamiana* . The Plant Cell, 31, 2456–2474. 31266900 10.1105/tpc.19.00099 PMC6790086

[plb70211-bib-0024] Halder V. , Suliman M.N.S. , Kaschani F. , Kaiser M. (2019) Plant chemical genetics reveals colistin sulphate as a SA and NPR1‐independent PR1 inducer functioning *via* a p38‐like kinase pathway. Scientific Reports, 9, 11196. 31371749 10.1038/s41598-019-47526-5 PMC6671972

[plb70211-bib-0025] Harberd N.P. (2003) Botany. Relieving DELLA restraint. Science, 299, 1853–1854. 12649470 10.1126/science.1083217

[plb70211-bib-0026] Heese A. , Hann D.R. , Gimenez‐Ibanez S. , Jones A.M. , He K. , Li J. , Schroeder J.I. , Peck S.C. , Rathjen J.P. (2007) The receptor‐like kinase SERK3/BAK1 is a central regulator of innate immunity in plants. Proceedings of the National Academy of Sciences of he United States of America, 104, 12217–12222. 10.1073/pnas.0705306104 PMC1924592 17626179

[plb70211-bib-0027] Heil M. , Baldwin I.T. (2002) Fitness costs of induced resistance: emerging experimental support for a slippery concept. Trends in Plant Science, 7, 61–67. 11832276 10.1016/s1360-1385(01)02186-0

[plb70211-bib-0028] Huang S. , Balgi A. , Pan Y. , Li M. , Zhang X. , Du L. , Zhou M. , Roberge M. , Li X. (2016) Identification of methylosome components as negative regulators of plant immunity using chemical genetics. Molecular Plant, 9, 1620–1633. 27756575 10.1016/j.molp.2016.10.006

[plb70211-bib-0029] Huot B. , Yao J. , Montgomery B.L. , He S.Y. (2014) Growth‐defense tradeoffs in plants: a balancing act to optimize fitness. Molecular Plant, 7, 1267–1287. 24777989 10.1093/mp/ssu049 PMC4168297

[plb70211-bib-0030] Jacob F. , Vernaldi S. , Maekawa T. (2013) Evolution and conservation of plant NLR functions. Frontiers in Immunology, 4, 297. 24093022 10.3389/fimmu.2013.00297 PMC3782705

[plb70211-bib-0031] Joglekar S. , Suliman M. , Bartsch M. , Halder V. , Maintz J. , Bautor J. , Zeier J. , Parker J.E. , Kombrink E. (2018) Chemical activation of EDS1/PAD4 signaling leading to pathogen resistance in Arabidopsis. Plant and Cell Physiology, 59, 1592–1607. 29931201 10.1093/pcp/pcy106

[plb70211-bib-0032] Jones J.D. , Dangl J.L. (2006) The plant immune system. Nature, 444, 323–329. 17108957 10.1038/nature05286

[plb70211-bib-0033] Kamatham S. , Pallu R. , Pasupulati A.K. , Singh S.S. , Gudipalli P. (2017) Benzoylsalicylic acid derivatives as defense activators in tobacco and Arabidopsis. Phytochemistry, 143, 160–169. 28818753 10.1016/j.phytochem.2017.07.014

[plb70211-bib-0034] Kempel A. , Schadler M. , Chrobock T. , Fischer M. , van Kleunen M. (2011) Tradeoffs associated with constitutive and induced plant resistance against herbivory. Proceedings of the National Academy of Sciences of he United States of America, 108, 5685–5689. 10.1073/pnas.1016508108 PMC3078369 21389269

[plb70211-bib-0035] Krumm T. , Bandemer K. , Boland W. (1995) Induction of volatile biosynthesis in the lima bean (*Phaseolus lunatus*) by leucine‐ and isoleucine conjugates of 1‐oxo‐ and 1‐hydroxyindan‐4‐carboxylic acid: evidence for amino acid conjugates of jasmonic acid as intermediates in the octadecanoid signalling pathway. FEBS Letters, 377, 523–529. 8549790 10.1016/0014-5793(95)01398-9

[plb70211-bib-0036] Kubota H. , Kakefuda A. , Watanabe T. , Ishii N. , Wada K. , Masuda N. , Sakamoto S. , Tsukamoto S. (2003) Synthesis and pharmacological evaluation of 1‐oxo‐2‐(3‐piperidyl)‐ 1,2,3,4‐ tetrahydroisoquinolines and related analogues as a new class of specific bradycardic agents possessing if channel inhibitory activity. Journal of Medicinal Chemistry, 46, 4728–4740. 14561092 10.1021/jm0301742

[plb70211-bib-0037] Lapin D. , Kovacova V. , Sun X. , Dongus J.A. , Bhandari D. , von Born P. , Bautor J. , Guarneri N. , Rzemieniewski J. , Stuttmann J. , Beyer A. , Parker J.E. (2019) A coevolved EDS1‐SAG101‐NRG1 module mediates cell death signaling by TIR‐domain immune receptors. The Plant Cell, 31, 2430–2455. 31311833 10.1105/tpc.19.00118 PMC6790079

[plb70211-bib-0038] Lepri A. , Longo C. , Messore A. , Kazmi H. , Madia V.N. , di Santo R. , Costi R. (2023) Plants and small molecules: an up‐ and coming synergy. Plants, 12, 1729. 37111951 10.3390/plants12081729 PMC10145415

[plb70211-bib-0039] Li J. , Wen J. , Lease K.A. , Doke J.T. , Tax F.E. , Walker J.C. (2002) BAK1, an Arabidopsis LRR receptor‐like protein kinase, interacts with BRI1 and modulates brassinosteroid signaling. Cell, 110, 213–222. 12150929 10.1016/s0092-8674(02)00812-7

[plb70211-bib-0040] Li L.‐L. , Xiao Y. , Wang B. , Zhuang Y. , Chen Y. , Lu J. , Lou Y. , Li R. (2024) A frameshift mutation in *JAZ10* resolves the growth versus defense dilemma in rice. Proceedings of the National Academy of Sciences of he United States of America, 121, e2413564121. 10.1073/pnas.2413564121 PMC11670064 39693337

[plb70211-bib-0041] Li X. , Kapos P. , Zhang Y. (2015) NLRs in plants. Current Opinion in Immunology, 32, 114–121. 25667191 10.1016/j.coi.2015.01.014

[plb70211-bib-0042] Li Y. , Yang Y. , Hu Y. , Liu H. , He M. , Yang Z. , Kong F. , Liu X. , Hou X. (2019) DELLA and EDS1 form a feedback regulatory module to fine‐tune plant growth‐defense tradeoff in Arabidopsis. Molecular Plant, 12, 1485–1498. 31382023 10.1016/j.molp.2019.07.006

[plb70211-bib-0043] Lozano‐Duran R. , Zipfel C. (2015) Trade‐off between growth and immunity: role of brassinosteroids. Trends in Plant Science, 20, 12–19. 25278266 10.1016/j.tplants.2014.09.003

[plb70211-bib-0044] Macho A.P. , Zipfel C. (2015) Targeting of plant pattern recognition receptor‐triggered immunity by bacterial type‐III secretion system effectors. Current Opinion in Microbiology, 23, 14–22. 25461568 10.1016/j.mib.2014.10.009

[plb70211-bib-0045] Margalha L. , Confraria A. , Baena‐Gonzalez E. (2019) SnRK1 and TOR: modulating growth‐defense trade‐offs in plant stress responses. Journal of Experimental Botany, 70, 2261–2274. 30793201 10.1093/jxb/erz066

[plb70211-bib-0046] Meister G. , Fischer U. (2002) Assisted RNP assembly: SMN and PRMT5 complexes cooperate in the formation of spliceosomal UsnRNPs. The EMBO Journal, 21, 5853–5863. 12411503 10.1093/emboj/cdf585 PMC131082

[plb70211-bib-0047] Monaghan J. , Zipfel C. (2012) Plant pattern recognition receptor complexes at the plasma membrane. Current Opinion in Plant Biology, 15, 349–357. 22705024 10.1016/j.pbi.2012.05.006

[plb70211-bib-0048] Monte I. , Hamberg M. , Chini A. , Gimenez‐Ibanez S. , Garcia‐Casado G. , Porzel A. , Pazos F. , Boter M. , Solano R. (2014) Rational design of a ligand‐based antagonist of jasmonate perception. Nature Chemical Biology, 10, 671–676. 24997606 10.1038/nchembio.1575

[plb70211-bib-0049] Nam K.H. , Li J. (2002) BRI1/BAK1, a receptor kinase pair mediating brassinosteroid signaling. Cell, 110, 203–212. 12150928 10.1016/s0092-8674(02)00814-0

[plb70211-bib-0050] Noutoshi Y. , Okazaki M. , Kida T. , Nishina Y. , Morishita Y. , Ogawa T. , Suzuki H. , Shibata D. , Jikumaru Y. , Hanada A. , Kamiya Y. , Shirasu K. (2012) Novel plant immune‐priming compounds identified *via* high‐throughput chemical screening target salicylic acid glucosyltransferases in Arabidopsis. Plant Cell, 24, 3795–3804. 22960909 10.1105/tpc.112.098343 PMC3480303

[plb70211-bib-0051] Noutoshi Y. , Shirasu K. (2018) A high‐throughput chemical screening method for inhibitors and potentiators of hypersensitive cell death using suspension cell culture of *Arabidopsis thaliana* . Methods in Molecular Biology, 1795, 39–47. 29846917 10.1007/978-1-4939-7874-8_4

[plb70211-bib-0052] Oh H.S. , Park D.H. , Collmer A. (2010) Components of the Pseudomonas syringae type III secretion system can suppress and may elicit plant innate immunity. Molecular Plant‐Microbe Interactions, 23, 727–739. 20459312 10.1094/MPMI-23-6-0727

[plb70211-bib-0053] Park S.Y. , Fung P. , Nishimura N. , Jensen D.R. , Fujii H. , Zhao Y. , Lumba S. , Santiago J. , Rodrigues A. , Chow T.F. , Alfred S.E. , Bonetta D. , Finkelstein R. , Provart N.J. , Desveaux D. , Rodriguez P.L. , McCourt P. , Zhu J.K. , Schroeder J.I. , Volkman B.F. , Cutler S.R. (2009) Abscisic acid inhibits type 2C protein phosphatases *via* the PYR/PYL family of START proteins. Science, 324, 1068–1071. 19407142 10.1126/science.1173041 PMC2827199

[plb70211-bib-0054] Peng Y. , Yang J. , Li X. , Zhang Y. (2021) Salicylic acid: biosynthesis and signaling. Annual Review of Plant Biology, 72, 761–791. 10.1146/annurev-arplant-081320-092855 33756096

[plb70211-bib-0055] Pieterse C.M. , Leon‐Reyes A. , van der Ent S. , van Wees S.C. (2009) Networking by small‐molecule hormones in plant immunity. Nature Chemical Biology, 5, 308–316. 19377457 10.1038/nchembio.164

[plb70211-bib-0056] Pluharova K. , Leontovycova H. , Stoudkova V. , Popsichalova R. , Marsik P. , Kloucek P. , Stardubtseva A. , Iakovenko O. , Krckova Z. , Valentova O. , Burketova L. , Janda M. , Kalanchova T. (2019) “Salicylic acid mutant collection” as a tool to explore the role of salicylic acid in regulation of plant growth under a changing environment. International Journal of Molecular Sciences, 20, 6365. 31861218 10.3390/ijms20246365 PMC6941003

[plb70211-bib-0057] Pluskota W.E. , Qu N. , Maitrejean M. , Boland W. , Baldwin I.T. (2007) Jasmonates and its mimics differentially elicit systemic defence responses in *Nicotiana attenuata* . Journal of Experimental Botany, 58, 4071–4082. 18065767 10.1093/jxb/erm263

[plb70211-bib-0058] Purrington C.B. (2000) Costs of resistance. Current Opinion in Plant Biology, 3, 305–308. 10873846 10.1016/s1369-5266(00)00085-6

[plb70211-bib-0059] Qi Y. , Wu J. , Yang Z. , Li H. , Sun X. , Wu X. , Nie J. , Zhou J. , Xu M. , Wu X. , Breen S. , Yu R. , Cheng D. , Sun Q. , Qiu H. , Zuo Y. , Boevink P.C. , Birch P.R.J. , Tian Z. (2024) Chloroplats elongation factors break the grwoth‐immunity trade‐off by simhltaneously promoting yield and defence. Nature Plants, 10, 1576–1591. 39300323 10.1038/s41477-024-01793-x

[plb70211-bib-0060] Ray N.C. , Bull R.J. , Finch H. , Heuvel M.V.D. , Bravo J.A. (2008) Oxazole and thiazole derivatives and their uses. WO 2008/096093 A1.

[plb70211-bib-0061] Sangster T.A. , Bahraqmi A. , Wilczek A. , Schellenberg K. , Kelley A. , Kong S.W. , Queitsch C. , Lindquist S. (2007) Phenotypic diversity and altered environmental plasticity in *Arabidopsis thaliana* with reduced Hsp90 levels. PLoS One, 2, e648. 17653275 10.1371/journal.pone.0000648 PMC1920555

[plb70211-bib-0062] Schreiber K. , Ckurshumova W. , Peek J. , Desveaux D. (2008) A high‐throughput chemical screen for resistance to Pseudomonas syringae in Arabidopsis. The Plant Journal, 54, 522–531. 18248597 10.1111/j.1365-313X.2008.03425.x

[plb70211-bib-0063] Serrano M. , Robatzek S. , Torres M. , Kombrink E. , Somssich I.E. , Robinson M. , Schulze‐Lefert P. (2007) Chemical interference of pathogen‐associated molecular pattern‐triggered immune responses in Arabidopsis reveals a potential role for fatty‐acid synthase type II complex‐derived lipid signals. Journal of Biological Chemistry, 282, 6803–6811. 17166839 10.1074/jbc.M608792200

[plb70211-bib-0064] Shen E. , Zhao T. , Zhu Q.‐H. (2024) Are miRNAs applicable for balancing crop growth and defense trade‐off. New Phytologist, 243, 1670–1680. 38952260 10.1111/nph.19939

[plb70211-bib-0065] Shigenaga A.M. , Argueso C.T. (2016) No hormone to rule them all: interactions of plant hormones during the responses of plants to pathogens. Seminars in Cell and Developmental Biology, 56, 174–189. 27312082 10.1016/j.semcdb.2016.06.005

[plb70211-bib-0066] Silverman F.P. , Petracek P.D. , Heiman D.F. , Fledderman C.M. , Warrior P. (2005) Salicylate activity. 3. Structure relationship to systemic acquired resistance. Journal of Agricultural and Food Chemistry, 53, 9775–9780. 16332130 10.1021/jf051383t

[plb70211-bib-0067] Simms E.L. , Triplett J. (1994) Costs and benefits of plant responses to disease: resistance and tolerance. Evolution, 48, 1973–1985. 28565152 10.1111/j.1558-5646.1994.tb02227.x

[plb70211-bib-0068] Sun B. , Huang J. , Kong L. , Gao C. , Zhao F. , Shen J. , Wang T. , Li K. , Wang L. , Wang Y. , Halterman D.A. , Dong S. (2024) Alternative splicing of a potato disease resistance gene maintains homeostasis between growth and immunity. The Plant Cell, 36, 3729–3750. 38941447 10.1093/plcell/koae189 PMC11371151

[plb70211-bib-0069] Sun X. , Lapin D. , Feehan J.M. , Stolze S.C. , Kramer K. , Dongus J.A. , Rzemieniewski J. , Blanvillain‐Baufume S. , Harzen A. , Bautor J. , Derbyshire P. , Menke F.L.H. , Finkemeier I. , Nakagami H. , Jones J.D.G. , Parker J.E. (2021) Pathogen effector recognition‐dependent association of NRG1 with EDS1 and SAG101 in TNL receptor immunity. Nature Communications, 12, 3335. 10.1038/s41467-021-23614-x PMC8185089 34099661

[plb70211-bib-0070] Sun Y. , Zhu Y.X. , Balint‐Kurti P.J. , Wang G.F. (2020) Fine‐tuning immunity: players and regulators for plant NLRs. Trends in Plant Science, 25, 695–713. 32526174 10.1016/j.tplants.2020.02.008

[plb70211-bib-0071] Tian D. , Traw M.B. , Chen J.Q. , Kreitman M. , Bergelson J. (2003) Fitness costs of R‐gene‐mediated resistance in *Arabidopsis thaliana* . Nature, 423, 74–77. 12721627 10.1038/nature01588

[plb70211-bib-0072] Todesco M. , Balasubramanian S. , Hu T.T. , Traw M.B. , Horton M. , Epple P. , Kuhns C. , Sureshkumar S. , Schwartz C. , Lanz C. , Laitinen R.A. , Huang Y. , Chory J. , Lipka V. , Borevitz J.O. , Dangl J.L. , Bergelson J. , Nordborg M. , Weigel D. (2010) Natural allelic variation underlying a major fitness trade‐off in Arabidopsis thaliana. Nature, 465, 632–636. 20520716 10.1038/nature09083 PMC3055268

[plb70211-bib-0073] Tsuchiya Y. , Vidaurre D. , Toh S. , Hanada A. , Nambara E. , Kamiya Y. , Yamaguchi S. , McCourt P. (2010) A small‐molecule screen identifies new functions for the plant hormone strigolactone. Nature Chemical Biology, 6, 741–749. 20818397 10.1038/nchembio.435

[plb70211-bib-0074] Tsuda K. , Sato M. , Stoddard T. , Glazebrook J. , Katagiri F. (2009) Network properties of robust immunity in plants. PLoS Genetics, 5, e1000772. 20011122 10.1371/journal.pgen.1000772 PMC2782137

[plb70211-bib-0075] Wagner S. , Stuttmann J. , Rietz S. , Guerois R. , Brunstein E. , Bautor J. , Niefind K. , Parker J.E. (2013) Structural basis for signaling by exclusive EDS1 heteromeric complexes with SAG101 or PAD4 in plant innate immunity. Cell Host & Microbe, 14, 619–630. 24331460 10.1016/j.chom.2013.11.006

[plb70211-bib-0076] Wasternack C. (2017) A plant's balance of growth and defense – revisited. New Phytologist, 215, 1291–1294. 28771818 10.1111/nph.14720

[plb70211-bib-0077] Wildermuth M.C. , Dewdney J. , Wu G. , Ausubel F.M. (2001) Isochorismate synthase ids required to synthesize salicylic acid for plant defence. Nature, 414, 562–565. 11734859 10.1038/35107108

[plb70211-bib-0078] Xiao J. , Nakamura Y. , Wu Z. , Fu W. , Chen Y. , Lou Y. , Baldwin I.T. , Boland W. , Li R. (2025) A synthetic jasmonate receptor agonist uncouples the growth‐defense trade‐off in rice. Proceedings of the National Academy of Sciences of he United States of America, 122, e2505675122. 10.1073/pnas.2505675122 PMC12184649 40493190

[plb70211-bib-0079] Yang H. , Shi Y. , Liu J. , Guo L. , Zhang X. , Yang S. (2010) A mutant CHS3 protein with TIR‐NB‐LRR‐LIM domains modulates growth, cell death and freezing tolerance in a temperature‐dependent manner in Arabidopsis. The Plant Journal, 63, 283–296. 20444230 10.1111/j.1365-313X.2010.04241.x

[plb70211-bib-0080] Zhao Y. , Chow T.F. , Puckrin R.S. , Alfred S.E. , Korir A.K. , Larive C.K. , Cutler S.R. (2007) Chemical genetic interrogation of natural variation uncovers a molecule that is glycoactivated. Nature Chemical Biology, 3, 716–721. 17891152 10.1038/nchembio.2007.32

[plb70211-bib-0081] Zhou M. , Wang W. (2018) Recent advances in synthetic chemical inducers of plant immunity. Frontiers in Plant Science, 9, 1613. 30459795 10.3389/fpls.2018.01613 PMC6232518

