# Supplementary material for: Chemical treatment rescues reduced growth of the autoimmune mutant chs3‐2D without compromising its immune responses
Source: Plant Biol (Stuttg). 2026 Mar 22;28(5):1686–97. doi: 10.1111/plb.70211 (PMC13358709; doi:10.1111/plb.70211)
Supplement: Supplementary file 1 — Table S1. Primers used for PCR experiments. Table S2. Starting compounds for synthesis of Ro 8‐4304 derivatives. Fig. S1. Synthesis of derivatives of Ro 8‐4304. Fig. S2. The relative fresh weight of chs3‐2D treated with different concentrations of DMSO, Ro 8‐4304, 4‐oxiranyl methoxybenzamide, and PTP‐F, as well as the derivatives Ro‐A01 to Ro‐A18. Fig. S3. Relative expression of the NIMIN1 gene in chs3‐2D seedlings grown at 18 °C for 21 days and treated with the respective compound at a concentration of 15 μM. [file PLB-28-1686-s001.pdf]

Supporting materials and methods

Synthesized compounds

Building bocks (BB)

BB01; 4-oxiranylmethoxy benzamide

The synthesis of 4-oxiranylmethoxy benzamide was done according to US patent no. 3,674,799, (1972)[1]. 4-hydroxybenzamide (3 mmol) [Fluorochem, Hadfield, United Kingdom] was added to a solution of sodium hydroxide (NaOH) (3 mmol) [Grüssing GmbH, Filsum, Germany] in 5 ml of water (H_2O). The mixture was treated with epichlorohydrine (9 mmol) [J&K Scientific GmbH, Pforzheim, Germany], and stirred at room temperature (RT) overnight (O/N). The precipitated 4-oxiranylmethoxy benzamide was filtered, and washed with H_2O. The compound was retrieved in a yield of 499 mg (86%), and was used without further purification.

HRMS *m/z* (ESI) 194.082, consistent with empirical formula $C_{10}H_{11}NO_3$ with an accuracy of 0.0081 ppm (accepted as $[M+H]^+$).

^{1}H NMR: (500 MHz, MeOH-d_4) δ 7.90-7.83 (m, 2H), 7.10-7.01 (m, 2H), 4.41 (dd, *J*=11.3, 2.5 Hz, 1H), 3.95 (dd, *J*=11.4, 6.2 Hz, 1H), 3.38 (ddt, *J*=6.7, 4.4, 2.6 Hz, 1H), 2.91 (dd, *J*=5.0, 4.2 Hz, 1H), 2.78 (dd, *J*=5.0, 2.7 Hz, 1H).

^{13}C NMR: (101 MHz, MeOH- d_4) δ 130.67, 115.35, 70.39, 49.85, 49.54.

BB02; (4-(4-bromophenyl)-1,2,3,6-tetrahydropyridine)

Method 1

The synthesis of 4-(4-bromophenyl)-1,2,3,6-tetrahydropyridine was done according to Conway et al. (2012)[2]. 4-(4-bromopenyl-4-piperidinol) (1g, 3.9 mmol) [Sigma-Aldrich Chemie GmbH, Taufkirchen, Germany], and 30 ml 6 M aqueous HCl [VWR International GmbH, Darmstadt, Germany] were mixed, and heated at reflux (~85°C) O/N. The solution was allowed to slowly cool down to RT, and this was left alone over the day. Crystals were formed. These were filtered, washed with H_2O, diethyl ether, and dried O/N on the vacuum pomp. This yielded 313 mg (34%) of pink crystals. So, the H_2O layer was checked, by NMR. Also, this layer contained (clean) product, although this was probably not pure enough for crystallisation. The H_2O was removed yielding 515 mg (56%) of a yellowish solid.

Method 2

Another method was tried, according to Junker et al. (2016)[3]. 3 ml trifluoroacetic acid (TFA) [Sigma-Aldrich Chemie GmbH] was placed in a round-bottom flask and 1 g 4-(4-bromophenyl-4-piperidinol) (3.9 mmol) [Sigma-Aldrich Chemie GmbH] was added. The mixture was stirred for 4-5 h at ~90°C. The solution was allowed to cool down to RT, and the TFA was removed, in vacuo, in combination with methanol (MeOH). This left a yellowish powder in a quantitative amount.

HRMS *m/z* (ESI) 238.022, consistent with empirical formula $C_{11}H_{12}BrN$ with an accuracy of 0.0067 ppm (accepted as $[M+H]^+$).

^{1}H NMR (500 MHz, DMSO-d_6) δ 9.36 (s, 2H), 7.62–7.55 (m, 2H), 7.48–7.41 (m, 2H), 6.25 (tt, *J*=3.5, 1.6 Hz, 1H), 3.73 (q, *J*=2.4 Hz, 2H), 3.29 (t, *J*=6.0 Hz, 2H), 2.67 (tq, *J*=6.3, 2.0 Hz, 2H).

^{13}C NMR (126 MHz, DMSO-d_6) δ 138.32, 133.62, 131.83, 127.26, 121.38, 118.15, 41.73, 40.28, 23.30.

BB03; (4-(4-hydroxy-phenyl)-1,2,3,6-tetrahydropyridine)

Method 1

The synthesis of 4-(4-hydroxy-phenyl)-1,2,3,6-tetrahydropyridine was done according to Gessner et al. (1985)[4]. 4-piperidone monohydrate (2 mmol) [Apollo Scientific, Stockport, United Kingdom] and phenol (2 mmol) [Grüssing GmbH] were dissolved in 5 ml of glacial acetic acid (AcOH) [VWR International GmbH]. Gaseous HCl was passed through the solution for 3 min, according to Arnáiz (1995)[5]. The mixture was heated to reflux (~85°C) for 1 h, and the mixture was allowed to cool down to RT. Gaseous HCl was passed again through the solution for 3 min, and the mixture was re-heated to reflux for 1 h. The mixture was allowed to cool down to RT. The AcOH was removed in vacuo giving a yellow/orange solid. This was purified in reverse phase on a flash chromatograph with an eluent of 100% H_2O to 100% acetonitrile (ACN). Appropriate fractions were combined and the solvent of these fractions was removed in vacuo, leaving a colorless powder in a yield of 65 mg (21%).

Method 2

4-piperidone monohydrate (2 mmol) [Apollo Scientific], and phenol (2 mmol) [Grüssing GmbH] were dissolved in 5 ml of ethanolic HCl (1M). The mixture was heated to reflux (~85°C). After two hours, 2 ml ethanolic HCl was added, and this was repeated three times with 1 h intervals, the next day this was again repeated 3 times. So, in total 12 ml ethanolic HCl was added at six different time points. On day two, the heating was stopped and the mixture was allowed to cool down to RT. The ethanolic HCl was removed in vacuo giving a yellow/orange solid. The product was purified in reverse phase on a flash chromatograph with an eluent of 100% H_2O to 100% ACN. Appropriate fractions were combined and the solvent of these fractions was removed in vacuo, leaving a colorless powder in a yield of 143 mg (41%).

HRMS *m/z* (ESI) 176.106, consistent with empirical formula $C_{11}H_{13}NO$ with an accuracy of 0.0003 ppm (accepted as $[M+H]^+$).

^{1}H NMR: (500 MHz, D_2O) δ 7.32 (d, *J*=8.2 Hz 2H), 6.82 (d, *J*=8.2 Hz, 2H), 5.95 (dq, *J*=3.8, 1.7 Hz, 1H), 3.74 (q, *J*=2.6 Hz, 2H, H-7), 3.38 (t, *J*=6.2 Hz, 2H), 2.67 (td, *J*=6.3, 3.5 Hz, 2H).

^{13}C NMR: (101 MHz, D_2O) δ 155.57, 134.46, 131.32, 126.56, 115.44, 113.97, 42.13, 40.97, 23.23.

BB04; 4-phenyl-1,2,3,6-tetrahydropyridine

The synthesis of 4-phenyl-1,2,3,6-tetrahydropyridine was done according to **Method 2**, described in **BB02**. 3 ml TFA [Sigma-Aldrich Chemie GmbH] was placed in a round-bottom flask and 1 g 4-phenyl-4-piperidinol (5.6 mmol) [Sigma-Aldrich Chemie GmbH] was added. The mixture was stirred overnight at ~90°C. The solution was allowed to cool down to RT, and the TFA was removed, in vacuo, in combination with MeOH. This left beige crystals in quantitative amounts.

HRMS *m/z* (ESI) 160.113, consistent with empirical formula $C_{11}H_{13}N$ with an accuracy of 0.0082 ppm (accepted as $[M+H]^+$).

^{1}H NMR (400 MHz, MeOH-d_4) δ 7.52–7.45 (m, 2H), 7.44–7.34 (m, 2H), 7.38–7.29 (m, 1H), 6.17 (tt, *J*=3.5, 1.7 Hz, 1H), 3.87 (dt, *J*=3.6, 2.5 Hz, 2H), 3.49 (t, *J*=6.1 Hz, 2H), 2.83 (tq, *J*=5.6, 2.0 Hz, 2H).

^{13}C NMR (101 MHz, MeOH-d_4) δ 139.01, 135.77, 128.30, 127.89, 124.76, 115.58, 42.09, 40.85, 23.51.

BB05; 4-[4-(trifluoromethyl)-phenyl]-1,2,3,6-tetrahydropyridine

The synthesis of 4-[4-(trifluoromethyl)-phenyl]-1,2,3,6-tetrahydropyridine was done according to **Method 2**, described in **BB02**. 2 ml TFA [Sigma-Aldrich Chemie GmbH] was placed in a round-bottom flask and 0.1549 g 4-[4-(trifluoromethyl)phenyl]4-piperidinol (0.63 mmol) [Matrix Scientific, Planegg, Germany] was added. The mixture was stirred for 2.5 h at ~90°C. The solution was allowed to cool down to rt, and the TFA was removed, in vacuo, in combination with MeOH. This resulted a dark orange oil in quantitative amounts.

HRMS *m/z* (EI) 228.100, consistent with empirical formula $C_{12}H_{12}F_3N$ with an accuracy of 0.0078 ppm (accepted as $[M+H]^+$).

^{1}H NMR (500 MHz, DMSO-d_6) δ 7.85–7.71 (m, 6H), 6.43 (s, 0H), 3.36 – 3.30 (m, 2H), 2.25 – 2.15 (m, 2H), 1.84 (d, *J*=14.1 Hz, 2H).

^{13}C NMR (126 MHz, DMSO-d_6) δ 159.10, 158.76, 158.48, 153.13, 127.86, 125.97, 125.87, 125.60, 125.56, 125.53, 125.49, 120.00, 68.72, 42.05, 34.48, 23.41.

^{19}F NMR: (565 MHz, DMSO-d_6) δ -77.64 (3F).

BB06; tert-butyl(4-hydroxybenzyl)carbamate

4-aminomethyl phenol (2.5 mmol) [S Sigma-Aldrich Chemie GmbH] was suspended in 10 ml dichloromethane (DCM). 2.5 Eq triethylamine (TEA) (6.25 mmol) [Grüssing GmbH, and 1.2 Eq di-tert-butyl-dicarbonate (Boc_2O) (3 mmol) [Sigma-Aldrich Chemie GmbH] were added. Everything dissolved after 5 min of stirring, and the solution was stirred O/N at RT. The next day, the organic layer is washed with H_2O, a saturated NaCl solution, and H_2O. Hereafter, the organic layer was dried with Na_2SO_4, and evaporated in vacuo. The resulting oil was purified by column chromatography (eluent: Petroleum ether (PE)/ethyl acetate (EtOAc) 7/3), yielding 309 mg (55%) of product (colorless oil).

HRMS *m/z* (ESI) 246.110, consistent with empirical formula $C_{12}H_{17}NO_3$ with an accuracy of 0.000 ppm (accepted as $[M+Na]^+$).

^{1}H NMR: (400 MHz, $CDCl_3$) δ 7.04 (d, *J*=7.8 Hz, 2H), 6.74-6.66 (m, 2H), 4.74 (s, 1H), 4.15 (d, 2H), 1.38 (s, 9H).

^{13}C NMR: (101 MHz, $CDCl_3$) δ 209.15, 189.12, 179.39, 155.18, 130.73, 129.84, 128.97, 115.47, 54.86, 46.90, 44.22, 28.44.

BB07; 4-oxiranylmethoxy-[tert-Butyl(4-hydroxybenzyl)carbamate]

4-oxiranylmethoxy-[tert-Butyl(4-hydroxybenzyl)carbamate] was synthesized comparable to **BB01**. However, the solvent was changed to MeOH. **BB06** (2.5 mmol) was added to a solution of 1 Eq of NaOH (2.5 mmol) [Grüssing GmbH] in 5 ml of MeOH. The mixture was treated with 3 Eq of epichlorohydrine (7.5 mmol) [J&K Scientific], and stirred at RT O/N. The solvent was removed in vacuo. The residue was partitioned between DCM, and H_2O. The DCM layer was separated, washed three times with H_2O, and dried with Na_2SO_4, before the solvent was evaporated in vacuo. This was purified by column chromatography (eluent: DCM/EtOAc, 9/1). Colorless oil was obtained, which turned into a colorless solid in a couple of hours, in a yield of 203 mg (29%).

HRMS *m/z* (ESI) 302.136, consistent with empirical formula $C_{15}H_{21}NO_4$ with an accuracy of 0.0003 ppm (accepted as $[M+Na]^+$).

^{1}H NMR: (600 MHz, $CDCl_3$) δ 7.13 (d, *J*=8.1 Hz, 2H), 6.83-6.78 (m, 2H), 4.71 (s, 1H), 4.17 (d, J=5.8 Hz, 2H), 4.14 (dd, *J*=11.0, 3.2 Hz, 1H), 3.88 (dd, *J*=11.0, 5.6 Hz, 1H), 3.28 (ddt, *J*=5.8, 4.1, 2.9 Hz, 1H), 2.83 (dd, *J*=4.9, 4.1 Hz, 1H), 2.68 (dd, *J*=4.9, 2.6 Hz, 1H), 1.39 (s, 9H).

^{13}C CNMR: (101 MHz, DMSO-d_6) δ 157.82, 155.85, 131.66, 128.89, 114.75, 105.03, 93.02, 69.86, 68.81, 50.15, 45.96, 45.76, 44.72, 44.13, 29.70, 28.42.

BB08; 2-[(4-fluorophenoxy)methyl]-oxirane

2-[(4-fluorophenoxy)methyl]-oxirane was synthesized comparable to **BB01**. 4-fluorophenol (2 mmol) [Grüssing GmbH] was added to a solution of 1 Eq of NaOH (2 mmol) [Grüssing GmbH] in 3 ml of H_2O. The mixture was treated with 3 Eq of epichlorohydrine (6 mmol) [J&K Scientific], and stirred at rt overnight. A yellow oil was visible in the H_2O layer. This was extracted into diethyl ether. The organic layer was separated, washed with a 5% NaOH solution, and H_2O. Then, the organic layer was dried with Na_2SO_4, and the solvent was evaporated in vacuo. The obtained clear yellow oil was purified by column chromatography (eluent: DCM/PE 8/2). The product, a colorless oil, was obtained in a yield of 165 mg (49%).

HRMS *m/z* (EI) 168.10, consistent with empirical formula $C_9H_9FO_2$ with an accuracy of 0.0413 ppm (accepted as $[M]^{\cdot +}$).

^{1}H NMR (600 MHz, $CDCl_3$) δ 6.94–6.87 (m, 2H), 6.83–6.76 (m, 2H), 4.13 (dd, *J*=11.0, 3.0 Hz, 1H), 3.84 (dd, *J*=11.0, 5.7 Hz, 1H), 3.27 (ddt, *J*=5.7, 4.1, 2.8 Hz, 1H), 2.84 (dd, *J*=4.9, 4.1 Hz, 1H), 2.68 (dd, *J*=4.9, 2.7 Hz, 1H).

^{13}C NMR (151 MHz, CDCl3) δ 158.33, 156.75, 154.64, 154.62, 115.96, 115.81, 115.78, 115.72, 69.46, 51.28, 46.96, 45.00, 44.64.

^{19}F NMR: (565 MHz, $CDCl_3$) δ -123.4 (1F).

BB09; (2-phenoxymethyl)-oxirane

(2-phenoxymethyl)-oxirane was synthesized comparable to **BB01**. Phenol (2 mmol) [Grüssing GmbH] was added to a solution of 1 Eq of NaOH (2 mmol) [Grüssing GmbH] in 3 ml of H_2O. The mixture was treated with 3 Eq of epichlorohydrine (6 mmol) [J&K Scientific], and stirred at RT O/N. A colorless oil was visible in the H_2O layer. This was extracted into diethyl ether. The organic layer was separated, washed with a 5% NaOH solution, and H_2O. Then, the organic layer was dried with Na_2SO_4, and the solvent was evaporated in vacuo. The obtained colorless oil was purified by column chromatography (eluent: DCM/PE, 8/2). The product, a colorless oil, was obtained in a yield of 127 mg (42%).

HRMS *m/z* (EI), 150.12, consistent with empirical formula $C_9H_{10}O_2$ with an accuracy of 0.0519 ppm (accepted as $[M]^{\cdot +}$).

^{1}H NMR (600 MHz, $CDCl_3$) δ 7.26–7.19 (m, 2H), 6.90 (tt, *J*=7.4, 1.1 Hz, 1H), 6.88–6.83 (m, 2H), 4.14 (dd, *J*=11.0, 3.3 Hz, 1H), 3.90 (dd, *J*=11.0, 5.6 Hz, 1H), 3.29 (ddt, *J*=5.7, 4.0, 2.9 Hz, 1H), 2.84 (dd, *J*=4.9, 4.1 Hz, 1H), 2.69 (dd, *J*=4.9, 2.7 Hz, 1H).

^{13}C NMR (151 MHz, $CDCl_3$) δ 158.47, 129.53, 121.24, 114.63, 68.67, 68.40, 50.17, 44.78.

BB10; 4-oxiranylmethoxy-4-hydroxy acetophenon

4-oxiranylmethoxy-4-hydroxy acetophenon was synthesized comparable to **BB01**. 4-hydroxyacetophenon (3 mmol) [Merck, Darmstadt, Germany] was added to a solution of 1 Eq of NaOH (3 mmol) [Grüssing GmbH, DE] in 5 ml of H_2O. The mixture was treated with 3 Eq of epichlorohydrine (9 mmol) [J&K Scientific], and stirred at rt overnight. A colorless oil was visible in the H_2O layer. This was extracted into EtOAc. The organic layer was separated, washed twice with H_2O, and once with brine. Then, the organic layer was dried with Na_2SO_4, and the solvent was evaporated in vacuo. The product was purified by column chromatography (eluent: EtOAc/PE 5/5). A colorless oil was obtained in a yield of 490 mg (85%).

HRMS *m/z* (EI) 192.11, consistent with empirical formula $C_{11}H_{12}O_3$ with an accuracy of 0.0314 ppm (accepted as $[M]^{\cdot +}$).

^{1}H NMR (400 MHz, $CDCl_3$) δ 7.91–7.82 (m, 2H), 6.93–6.84 (m, 2H), 4.25 (dd, *J*=11.1, 3.0 Hz, 1H), 3.93 (dd, *J*=11.1, 5.8 Hz, 1H), 3.30 (ddt, *J*=5.7, 4.1, 2.8 Hz, 1H), 2.86 (dd, *J*=4.9, 4.1 Hz, 1H), 2.77–2.67 (m, 1H), 1.97 (s, 3H).

^{13}C NMR (126 MHz, $CDCl_3$) δ 197.17, 197.13, 196.27, 171.56, 162.69, 162.49, 131.30, 131.16, 131.04, 131.00, 114.68, 114.63, 70.06, 69.25, 69.09, 60.78, 50.29, 46.24, 44.97, 26.74, 21.43, 14.58.

BB11; 4-oxiranylmethoxy-4-ethyl phenol

4-oxiranylmethoxy-4-ethyl phenol was synthesized comparable to **BB01**. 4-ethylphenol (3 mmol) [Sigma-Aldrich Chemie GmbH] was added to a solution of 1 Eq of NaOH (3 mmol) [Grüssing GmbH] in 5 ml of H_2O. The mixture was treated with 3 Eq of epichlorohydrine (9 mmol) [J&K Scientific], and stirred at rt overnight. A colorless oil was visible in the H_2O layer. This was extracted into DCM. The organic layer was separated, washed twice with H_2O, and once with brine. Then, the organic layer was dried with Na_2SO_4, and the solvent was evaporated in vacuo. The obtained yellow oil was purified by column chromatography (eluent: DCM/PE 8/2). A clear yellow oil was obtained in a yield of 162 mg (30%).

HRMS *m/z* (EI) 178.11, consistent with empirical formula $C_{11}H_{14}O_2$ with an accuracy of 0.0106 ppm (accepted as $[M]^{\cdot +}$).

^{1}H NMR (500 MHz, $CDCl_3$) δ 7.18–7.11 (m, 2H), 6.91–6.85 (m, 2H), 4.21 (dd, *J*=11.0, 3.3 Hz, 1H), 3.98 (dd, *J*=11.1, 5.6 Hz, 1H), 3.37 (ddt, *J*=5.8, 4.0, 3.0 Hz, 1H), 2.93 (dd, *J*=5.0, 4.1 Hz, 1H), 2.78 (dd, *J*=5.0, 2.6 Hz, 1H), 2.62 (q, *J*=7.6 Hz, 2H), 1.24 (t, *J*=7.6 Hz, 3H).

^{13}C NMR (126 MHz, $CDCl_3$) δ 187.91, 156.97, 137.44, 129.17, 114.96, 69.27, 50.64, 45.18, 28.38, 16.24.

BB12; 4-(3-bromoethoxy)-benzamide

The synthesis 4-(3-bromoethoxy)-benzamide was done according to Kubota et al. (2003) and patent no. WO 2008096093/A1[6,7]. 4-hydroxybenzamide (3 mmol) [Fluorochem] was dissolved in 10 ml ACN. K_2CO_3 (1.5 Eq, 4.5 mmol) [Grüssing GmbH], and 1,2-dibromoethane (5 Eq, 15 mmol) [Merck] were added. This mixture was stirred at ~80°C overnight. The ACN was removed in vacuo, and the leftover product was participated between H_2O and EtOAc. The organic layer was separated, washed with H_2O, a 5% NaOH solution, H_2O, and brine. After this the organic layer was dried with Na_2SO_4, and the solvent was removed in vacuo. The resulting product was purified by column chromatography (eluents: EtOAc/MeOH 9.5/0.5), yielding 147 mg (30%) of product (colorless solid).

HRMS *m/z* (ESI) 244.000, consistent with empirical formula $C_9H_{10}BrNO_2$ with an accuracy of 0.0105 ppm (accepted as $[M+H]^+$).

^{1}H NMR (600 MHz, $CDCl_3$) δ 7.73 (d, *J*=8.5 Hz, 2H), 6.89 (d, *J*=8.4 Hz, 2H), 6.04 (s, 1H), 5.77 (s, 1H), 4.28 (t, *J*=6.2 Hz, 2H), 3.59 (t, *J*=6.2 Hz, 2H).

^{13}C NMR (151 MHz, $CDCl_3$) δ 129.51, 125.85, 114.53, 67.89, 28.62.

BB13; 4-(3-bromopropoxy)-benzamide

The synthesis of 4-(3-bromopropoxy)-benzamide was done as described in **BB12**. On a 5 mmol scale, using 1,3-dibromopropane (5 Eq, 25 mmol) [Alfa Aesar GmbH & Co KG, Karlsruhe, Germany], instead of 1,2-dibromoethane.The resulting product was purified by column chromatography (eluent: EtOAc), yielding 886 mg (69%) of product (colorless solid).

HRMS *m/z* (ESI) 258.995, consistent with empirical formula $C_{10}H_{12}BrNO_2$ with an accuracy of 0.9899 ppm (accepted as $[M+H]^+$).

^{1}H NMR (600 MHz, $CDCl_3$) δ 7.77–7.72 (m, 2H), 6.88 (d, *J*=8.4 Hz, 2H), 6.06 (s, 1H), 4.10 (t, *J*=5.8 Hz, 2H), 3.54 (t, *J*=6.4 Hz, 2H), 2.28 (h, *J*=6.1 Hz, 2H).

^{13}C NMR (151 MHz, $CDCl_3$) δ 207.58, 169.32, 162.10, 134.06, 129.59, 124.81, 115.21, 114.68, 114.44, 80.97, 65.65, 65.54, 34.87, 32.12, 31.11, 29.70, 29.49.

BB14; 4-(3-bromobutoxy)-benzamide

The synthesis of 4-(3-bromobutoxy)-benzamide was done as described in **BB12**. On a 3 mmol scale, using 1,4-dibromobutane (5 Eq, 15 mmol) [Merck], instead of 1,2-dibromoethane. The resulting product was purified twice by column chromatography (eluents: EtOAc/MeOH 9.5/0.5; EtOAc/MeOH 9.75/0.25), yielding 251 mg (31%) of product (colorless solid).

HRMS *m/z* (ESI) 272.007, consistent with empirical formula $C_{11}H_{14}BrNO_2$ with an accuracy of 0.0138 ppm (accepted as $[M+H]^+$).

^{1}H NMR (500 MHz, $CDCl_3$) δ 7.81–7.73 (m, 2H), 6.91–6.84 (m, 2H), 6.46 (s, 2H), 3.99 (t, *J*=6.0 Hz, 2H), 3.42 (t, *J*=6.5 Hz, 2H), 2.01 (dddd, *J*=12.0, 10.0, 6.1, 2.3 Hz, 2H), 1.98–1.87 (m, 2H).

^{13}C NMR (126 MHz, $CDCl_3$) δ 182.72, 176.55, 170.25, 130.17, 115.55, 114.90, 89.91, 77.67, 67.60, 33.63, 33.50, 28.12, 28.04.

BB15; 4-(3-bromopentoxy)-benzamide

The synthesis of 4-(3-bromopentoxy)-benzamide was done as described in **BB12**. On a 3 mmol scale, using 1,5-dibromopentane (5 E1, 15 mmol) [Alfa Aesar GmbH & Co KG], instead of 1,2-dibromoethane. The resulting product was purified by column chromatography (eluents: EtOAc/MeOH 9.5/0.5), yielding 498 mg (58%) of product (colorless solid).

HRMS *m/z* (ESI) 286.048, consistent with empirical formula $C_{12}H_{16}BrNO_2$ with an accuracy of 0.0122 ppm (accepted as $[M+H]^+$).

^{1}H NMR (400 MHz, $CDCl_3$) δ 7.84 (d, *J*=8.5 Hz, 2H), 6.96 (d, *J*=8.3 Hz, 2H), 6.41 (s, 2H), 4.05 (t, *J*=6.3 Hz, 2H), 3.48–3.43 (m, 2H), 2.03–1.93 (m, 2H), 1.87 (ddt, *J*=14.6, 8.3, 3.8 Hz, 2H), 1.73–1.60 (m, 2H).

^{13}C NMR (101 MHz, $CDCl_3$) δ 129.67, 115.16, 114.65, 114.46, 86.58, 78.79, 67.89, 33.49, 33.26, 32.40, 31.87, 24.76.

BB16; 4-oxiranylethoxy benzamide

4-oxiranylethoxy benzamide was synthesized comparable to **BB01**. 4-hydroxybenzamide (2 mmol) [Fluorochem] was added to a solution of 1 Eq of NaOH (2 mmol) [Grüssing GmbH] in 3 ml of H_2O. The mixture was treated with 3 Eq of 2-(2-chloroethyl)oxirane (6 mmol) [SIA Enamine, Riga, Latvia], and stirred at rt overnight. A colorless precipitate was visible, on the stirrer. This was filtered, scrapped off, and washed with H_2O, yielding 197 mg (48%) of a colorless solid.

HRMS *m/z* (EI) 246.071, consistent with empirical formula $C_{11}H_{13}NO_3$ with an accuracy of 0.0184 ppm (accepted as $[M+K]^+$).

^{1}H NMR (300 MHz, DMSO-d_6) δ 7.89–7.78 (m, 3H), 7.18 (s, 1H), 7.04–6.93 (m, 2H), 5.22–5.14 (m, 1H), 4.05–3.90 (m, 3H), 3.77 (ddd, *J*=7.9, 5.9, 1.7 Hz, 2H), 1.92 (tdd, *J*=14.1, 10.1, 5.9 Hz, 1H).

^{13}C NMR (75 MHz, DMSO-d_6) δ 167.85, 161.38, 129.80, 126.99, 114.38, 72.43, 65.99, 42.56, 36.96.

BB17; 4-[(2-methyl-2-oxiranyl)methoxy]-benzamide

4-[(2-methyl-2-oxiranyl)methoxy]-benzamide was synthesized comparable to **BB01**. 4-hydroxybenzamide (2 mmol) [Sigma-Aldrich Chemie GmbH] was added to a solution of 1 Eq of NaOH (2 mmol) [Fluorochem] in 3 ml of H_2O. The mixture was treated with 3 Eq of 2-(chloromethyl)-2-methyloxirane (6 mmol) [Fluorochem], and stirred at rt overnight. A colorless precipitate was visible. This was filtered, and washed with H_2O, yielding 228 mg (55%) of a colorless solid.

HRMS *m/z* (EI) 208.081, consistent with empirical formula $C_{11}H_{12}NO_3$ with an accuracy of 0.0085 ppm (accepted as $[M+H]^+$).

^{1}H NMR (400 MHz, MeOH-*d*$_4$) δ 7.90–7.81 (m, 2H), 7.08–6.99 (m, 2H), 4.23 (d, *J*=10.9 Hz, 1H), 3.98 (d, *J*=10.9 Hz, 1H), 2.90 (d, *J*=4.8 Hz, 1H), 2.76 (d, *J*=4.8 Hz, 1H), 1.48 (s, 3H).
^{13}C NMR (126 MHz, DMSO-*d*$_6$) δ 167.74, 167.70, 161.41, 161.04, 129.70, 127.18, 126.96, 114.38, 72.49, 71.77, 70.70, 55.58, 51.13, 22.49, 18.52.

Derivatives

Ro 8-4304; (4-{3-[4-fluoro-phenyl-3, 6-dihyrdro-1(2H)-pyridyl]-2-hydoxy-propoxy}-benzamide)

The synthesis of **Ro8-4304** was adapted from US patent no. 3,674,799, 1972[1]. 4-(4-fluorophenyl-1,2,3,6-tetrahydropyridine·HCL (**PTP-F**) (0.5 mmol) [Acros Organics, Geel, Belgium] was dissolved in 5 ml of ethanol (EtOH). 2 Eq (1 mmol) of TEA was added, and the mixture was stirred for 5 min before the addition of **BB01** (0.5 mmol). This mixture was stirred at reflux (~78°C) for 4 h. The reaction mixture was allowed to cool down, and a beige precipitate separated out. This was filtered, and washed with EtOH. The leftover product was treated with 3 ml ethanolic hydrochloric acid (1 M). This was stirred for 2 h, filtered, and washed with EtOH, giving 84 mg of beige compound (45.6%). The residual EtOH was removed in vacuo, and the product was used without further purification.

Ethanolic hydrochloric acid (1 M) is created by slowly adding acetyl chloride (0.71 ml) [Merck] to cooled EtOH (9.29 ml, ~0°C).

HRMS *m/z* (ESI) 371.178, consistent with empirical formula $C_{21}H_{23}FN_2O_3$ with an accuracy of 0.0087 ppm (accepted as $[M+H]^+$).
^{1}H NMR (600 MHz, DMSO-*d*$_6$) δ 10.54 (s, 0H), 10.43 (s, 1H), 7.90–7.83 (m, 3H), 7.59-7.52 (m, 2H), 7.27–7.20 (m, 2H), 7.21 (s, 1H), 7.05–6.99 (m, 2H), 6.19 (q, *J*=3.3, 2.9 Hz, 1H), 6.05 (s, 1H), 4.59–4.34 (m, 1H), 4.12 (d, *J*=16.4 Hz, 1H), 4.07 (d, *J*= 4.9 Hz, 2H), 3.95–3.88 (m, 1H), 3.80 (d, *J*=11.9 Hz, 1H), 3.68 (d, *J*=12.4 Hz, 1H), 3.49–3.39 (m, 1H), 3.31 (ddd, *J*=17.6, 13.3, 7.2 Hz, 1H), 2.95–2.87 (m, 1H), 2.76 (t, *J*=14.9 Hz, 1H).
^{13}C NMR (151 MHz, DMSO-*d*$_6$) δ 167.78, 163.16, 161.53, 161.06, 135.25, 135.23, 129.86, 127.42, 127.36, 116.73, 116.49, 115.91, 115.76, 114.46, 106.85, 70.67, 64.50, 64.29, 57.84, 57.72, 51.72, 50.00, 49.88, 48.50, 24.07, 23.75.
^{19}F NMR: (565 MHz, DMSO-*d*$_6$) δ -114.72 (1F).

Ro-A01; 4-[3-[4-(4-bromophenyl)-3,6-dihydro-1(2H)-pyridinyl]-2-hydroxypropoxy]-benzamide

Ro-A01 was synthesized on a 0.5 mmol scale using **BB01**, and **BB02**. The synthesis was performed as described in **Ro 8-4304**. However, the reaction was stirred at reflux overnight, and the work-up was changed. The precipitate could not be filtered after cooling down to RT, while it was too fine. So, the solvent was removed in vacuo, and the product was treated with 3 ml ethanolic HCl (1M). This was stirred for 3 hr, and the solvent was again removed in vacuo. The leftover compound dissolved in MeOH, but not in DCM, so recrystallization was tried, with two solvents. A light beige solid precipitated out of the yellow solution. This was filtered and washed with DCM. This gave the product in an amount of 79 mg (37%).
HRMS *m/z* (ESI) 431.0973, consistent with empirical formula $C_{21}H_{23}BrN_2O_3$ with an accuracy of 0.0081 ppm (accepted as $[M+H]^+$).
^{1}H NMR (400 MHz, DMSO-*d*$_6$) δ 7.90–7.81 (m, 3H), 7.63–7.57 (m, 2H), 7.51–7.42 (m, 2H), 7.20 (s, 1H), 7.05–6.98 (m, 2H), 6.27 (s, 1H), 4.44 (s, 1H), 4.06 (d, *J*=5.0

Hz, 2H), 3.88 (d, *J*=43.8 Hz, 1H), 3.00 (s, 3H), 2.97–2.59 (m, 2H), 1.21 (dt, *J*= 12.2, 7.0 Hz, 2H).
^{13}C NMR (101 MHz, DMSO-d_6) δ 167.78, 131.95, 129.86, 127.45, 114.46, 70.61, 49.06.

Ro-A02; (4-{3-[4-hydroxy-phenyl-3, 6-dihyrdro-1(2H)-pyridyl]-2-hydoxy-propoxy}-benzamide)
Ro-A02 was synthesized on a 0.3 mmol scale using **BB01**, and **BB03**. The synthesis was performed as described in **Ro 8-4304**. This reaction yielded 47 mg (42%) of a yellow solid. This was used without further purification.
HRMS *m/z* (ESI) 369.152, consistent with empirical formula $C_{21}H_{24}N_2O_4$ with an accuracy of 0.0216 ppm (accepted as $[M+H]^+$).
^{1}H NMR (400 MHz, DMSO-d_6) δ 10.33 (d, *J*=45.7 Hz), 7.86 (d, *J*=8.2 Hz, 2H), 7.32 (d, *J*=8.6 Hz, 2H), 7.23–7.19 (m, 2H), 7.01 (dt, *J*=9.0, 2.0 Hz, 2H), 6.78 (d, *J*=8.6 Hz, 2H), 6.02 (s, 1H), 4.69–4.38 (m, 1H), 4.06 (d, *J*=4.8 Hz, 2H), 3.47–3.26 (m, 2H), 3.05 (dd, *J*=7.3, 4.8 Hz, 1H), 2.98–2.58 (m, 2H), 1.20 (t, *J*=7.3 Hz, 1H), 1.05 (t, *J*=7.0 Hz, 2H).
^{13}C NMR (101 MHz, DMSO-d_6) δ 167.30, 160.55, 129.36, 125.94, 115.24, 113.95, 113.03, 112.80, 70.16, 63.99, 63.75, 45.33.

Ro-A03; [3-(3,6-dihydro-4-phenyl-1(2H)-pyridinyl)-2-hydroxypropoxy]-benzamide
Ro-A03 was synthesized on a 1 mmol scale using **BB01**, and **BB04**. The synthesis was performed as described in **Ro 8-4304**. However, the reaction was stirred at reflux O/N, and the work-up was changed. The next day, a red precipitate was visible, and the solution was slowly allowed to cool down to RT. Hereafter, the solution was treated with ethanolic HCl (1M) till the pH reached 2-3. This was stirred for 3 h. The precipitate re-appeared, and was filtered, washed with EtOH, and placed on the vacuum pump for drying. The beige product was obtained in an amount of 240 mg (68%).
HRMS *m/z* (ESI) 353.187, consistent with empirical formula $C_{21}H_{24}N_2O_3$ with an accuracy of 0.0083 ppm (accepted as $[M+H]^+$).
^{1}H NMR (500 MHz, DMSO-d_6) δ 7.91–7.82 (m, 2H), 7.85 (s, 1H), 7.54–7.49 (m, 2H), 7.44–7.37 (m, 2H), 7.38–7.31 (m, 1H), 7.20 (s, 1H), 7.06–6.98 (m, 2H), 6.22 (s, 1H), 6.01 (s, 1H), 4.43 (d, *J*=15.4 Hz, 1H), 4.17–4.03 (m, 3H), 3.94 (s, 1H), 3.85–3.66 (m, 1H), 3.60–3.57 (m, 1H), 3.08 (dd, *J*=7.3, 4.8 Hz, 0H), 2.93 (d, *J* = 11.4 Hz, 0H), 2.84–2.74 (m, 2H), 1.26–1.16 (m, 1H).
^{13}C NMR (126 MHz, DMSO-d_6) δ 167.67, 160.94, 129.76, 128.98, 128.38, 125.19, 114.36, 70.52, 61.68, 56.39, 46.00, 28.99, 18.92.

Ro-A04; 4-[3-[3,6-dihydro-1(2H)-pyridinyl]-2-hydroxypropoxy]-benzamide
Ro-A04 was synthesized on a 1 mmol scale using **BB01**, and 1,2,3,6-tetrahydropyridine.HCl [abcr GmbH & Co. KG, Karlsruhe, Germany]. The synthesis was performed as described in **Ro 8-4304**. However, the reaction was stirred at reflux O/N. The next day, this was slowly cooled down to RT. A yellow solution was visible; EtOH was removed in vacuo, and the product was purified in reverse phase on a flash chromatograph with an eluent of 100% H_2O to 100% ACN. Appropriate fractions were combined and the solvent of these fractions was removed in vacuo, leaving a colorless powder in a yield of 69 mg (25%).
HRMS *m/z* (ESI) 277.133, consistent with empirical formula $C_{15}H_{28}N_2O_3$ with an accuracy of 0.0144 ppm (accepted as $[M+H]^+$).
^{1}H NMR (500 MHz, DMSO-d_6) δ 7.87–7.80 (m, 2H), 7.82 (s, 1H), 7.16 (s, 1H), 7.01–6.95 (m, 2H), 5.73–5.60 (m, 2H), 4.90 (d, *J*=4.8 Hz, 1H), 4.05 (dd, *J*=9.7, 3.6 Hz,

1H), 4.02–3.96 (m, 1H), 3.93 (dd, *J*=9.7, 6.0 Hz, 1H), 3.32 (s, 1H), 3.00–2.94 (m, 2H), 2.64-2.52 (m, 2H), 2.44 (dd, *J*=12.7, 6.3 Hz, 1H), 2.08 (dh, *J*=7.5, 2.4 Hz, 2H).
^{13}C NMR (126 MHz, DMSO-d_6) δ 167.78, 161.49, 159.11, 129.70, 126.77, 126.02, 125.16, 114.26, 71.58, 66.92, 61.39, 53.32, 50.64, 26.13.

Ro-A05; 4-[3-[4-(4-trifluoromethylphenyl)-piperidin-4-ol]-2-hydroxypropoxy]-benzamide

Ro-A05 was synthesized on a 1 mmol scale using **BB01**, and [4-(trifluoromethyl)phenyl]4-piperidinol (0.88 mmol) [Matrix Scientific]. The synthesis was performed as described in **Ro 8-4304**. However, the reaction was stirred at reflux O/N. The next day, this was slowly cooled down to RT. An orange solution was visible. EtOH was removed in vacuo, and the obtained product was purified by column chromatography (eluent: EtOAc/MeOH 9/1). This left a beige solid, in a yield of 89 mg (21%).
HRMS *m/z* (ESI) 439.185, consistent with empirical formula $C_{22}H_{25}F_3N_2O_4$ with an accuracy of 0.0084 ppm (accepted as $[M+H]^+$).
^{1}H NMR (600 MHz, DMSO-d_6) δ 7.93–7.87 (m, 2H), 7.88 (s, 1H), 7.76 (d, *J*=8.4 Hz, 2H), 7.73 (d, *J*=8.5 Hz, 2H), 7.23 (s, 1H), 7.09–7.03 (m, 2H), 4.93 (s, 1H), 4.14 (dd, *J*=9.8, 3.4 Hz, 2H), 4.06 (s, 1H), 4.01 (dd, *J*=9.8, 6.1 Hz, 2H), 2.82 (s, 1H), 2.76 (s, 1H), 2.60 (s, 1H), 2.51 (s, 2H), 2.06–1.98 (m, 2H), 1.64 (dd, *J*=13.8, 6.3 Hz, 2H).
^{13}C NMR (151 MHz, DMSO-d_6) δ 167.87, 161.61, 129.95, 129.80, 127.30, 126.88, 126.21, 125.27, 125.24, 125.21, 114.40, 113.92, 104.44, 71.77, 70.16, 61.62, 58.51, 50.18, 40.52, 38.28.
^{19}F NMR: (565 MHz, DMSO-d_6) δ -60.79 (3F).

Ro-A06; 4-[3-[4-(4-trifluoromethylphenyl)-3,6-dihydro-1(2H)-pyridinyl]-2-hydroxypropoxy]-benzamide

R0-A06 was synthesized on a 0.6 mmol scale using **BB01,** and **BB05**. The synthesis was performed as described in **Ro 8-4308**. However, the reaction was stirred at reflux O/N. The next day, this was slowly cooled down to RT. An orange solution was visible. EtOH was removed in vacuo, and the obtained product was purified by column chromatography (eluent: EtOAc/MeOH 9/1). This left a yellow oil, in a yield of 16 mg (6%). This was still contaminated, however since the yield was so low, no further purification was done.
HRMS *m/z* (ESI) 421.171, consistent with empirical formula $C_{22}H_{23}F_3N_2O_3$ with an accuracy of 0.0049 ppm (accepted as $[M+H]^+$).
^{1}H NMR (600 MHz, DMSO-d_6) δ 7.89–7.81 (m, 2H), 7.71 (s, 2H), 7.70–7.64 (m, 2H), 7.19 (d, *J*=16.6 Hz, 2H), 7.04–6.95 (m, 2H), 6.35 (s, 1H), 4.22–4.10 (m, 1H), 4.12–4.06 (m, 2H), 3.97 (s, 1H), 3.17 (d, *J*=5.2 Hz, 1H), 2.74 (s, 1H), 1.99 (s, 4H).
^{13}C NMR (151 MHz, DMSO-d_6) δ 167.83, 161.33, 129.85, 129.83, 114.44, 114.40, 113.92, 69.80, 67.77, 60.22, 21.23, 14.56.
^{19}F NMR: (565 MHz, DMSO-d_6) δ -73.38 (3F).

Ro-A07 & Ro-A08; 4-[3-[4-(4-fluorophenyl)-3,6-dihydro-1(2H)-pyridinyl]-2-hydroxypropoxy]-benzylamine

Ro-A07 was synthesized on a 1 mmol scale using **BB07**, and **PTP-F**. The synthesis was performed as described in **Ro 8-4304**. However, the reaction was stirred at reflux O/N, and the work-up was changed. After refluxing, the solution was allowed to cool down to RT. EtOH was removed in vacuo, and a small amount of material was taken, this is **Ro-A08**. To the rest, 8 ml ethanolic HCl (1M) was added, and the solution was stirred at reflux for 6 h. The solution was slowly allowed to cool down to RT, and a yellow precipitate separated out. This was filtered, and washed with EtOH;

residual solvent was removed in vacuum O/N. A beige solid was obtained in a yield of 128 mg (36%). This was used without further purification.
HRMS Ro-A07 *m/z* (ESI) 357.1997, consistent with empirical formula $C_{21}H_{25}FN_2O_2$ with an accuracy of 0.0097 ppm (accepted as $[M+H]^+$).
HRMS Ro-A08 *m/z* (ESI) 479.2317, consistent with empirical formula $C_{26}H_{33}FN_2O_4$ with an accuracy of 0.0535 ppm (accepted as $[M+Na]^+$).
^{1}H NMR Ro-A07 (600 MHz, DMSO-d_6) δ 10.58 (s, 1H); 10.47 (s, 1H); 8.37 (s, 3H, H-1); 7.59–7.51 (m, 2H, H-4); 7.47–7.40 (m, 2H, H-14); 7.27–7.19 (m, 2H, H-3); 7.04–6.97 (m, 2H, H-13); 6.19 (d, *J*=3.8 Hz, 1H, H-12); 6.07 (s, 0H, H-8); 6.02 (s, 0H, H-8); 4.51–4.43 (m, 1H, H-6); 4.09 (dd, *J*=34.5, 16.5 Hz, 1H, H-5); 4.01 (d, *J*=5.0 Hz, 2H, H-11); 3.95 (q, *J*=5.9 Hz, 3H, H-2, H-5); 3.79 (d, *J*=12.4 Hz, 1H, H-7); 3.68 (dt, *J*=11.3, 3.4 Hz, 1H, H-7); 3.49–3.39 (m, 1H, H-10); 3.29 (ddd, *J*=13.9, 11.2, 3.8 Hz, 1H, H-10); 2.98–2.84 (m, 1H, H-9); 2.76 (t, *J*=13.5 Hz, 1H, H-9).
^{1}H NMR Ro-A08 (600 MHz, DMSO-d_6) δ 9.77 (s, 2H); 9.12 (s, 1H); 8.28 (s, 3H, H-1); 7.54 (ddd, *J*=15.9, 8.9, 5.5 Hz, 2H, H-4); 7.45–7.38 (m, 2H, H-14); 7.23 (q, *J*=8.8 Hz 2H, H-3); 7.04–6.98 (m, 2H, H-13); 6.19 (s, 1H, H-12); 4.51–4.43 (m, 1H, H-6); 4.14-4.02 (m, 1H, H-5); 4.05-3.97 (m, 2H, H-11); 3.95 (d, *J*=5.9 Hz, 2H, H-5, H-7); 3.74 (d, *J*=4.7 Hz, 1H, H-7); 3.45 (t, *J*=7.0 Hz, 1H, H-10); 3.07 (qd, *J*=7.3, 4.8 Hz, 2H, H-2); 2.76 (t, *J*=15.9 Hz, 1H, H-9); 2.67 (s, 1H, H-9; 1.19 (t, *J*=7.3 Hz, 9H, H-0).
^{13}C NMR Ro-A07 (151 MHz, DMSO-d_6) δ 161.53, 158.87, 135.22, 133.65, 133.35, 131.03, 127.41, 127.35, 126.88, 126.85, 116.73, 116.50, 115.92, 115.90, 115.78, 115.76, 115.07, 70.70, 64.54, 64.34, 57.87, 57.83, 51.64, 50.03, 49.80, 48.55, 45.84, 42.09, 24.05, 23.77, 8.91.
^{19}F NMR Ro-A07 & Ro-A08: (565 MHz, DMSO-d_6) δ -114.56 (1F).

Ro-A09; 4-[3-[4-(4-fluorophenyl)-3,6-dihydro-1(2H)-pyridinyl]-2-hydroxypropoxy]-fluorophenyl

Ro-A09 was synthesized on a 0.75 mmol scale using **BB08**, and **PTP-F**. The synthesis was performed as described in **Ro 8-4304**. However, the reaction was stirred at reflux O/N. The next day, this was slowly cooled down to RT, before placing it in the freezer O/N. A precipitate was visible. This was filtered and washed with EtOH. The obtained product was treated with 3 ml ethanolic HCl (1M), and this was stirred O/N. The ethanolic liquid was removed in vacuo, leaving a colorless solid, in a yield of 142 mg (55%). This was used without further purification.
HRMS *m/z* (ESI) 346.178, consistent with empirical formula $C_{20}H_{21}F_2NO_2$ with an accuracy of 0.024 ppm (accepted as $[M+H]^+$).
^{1}H NMR (400 MHz, DMSO-d_6) δ 10.38 (s, 1H), 10.28 (s, 1H), 7.66–7.56 (m, 2H), 7.29 (t, *J*=8.8 Hz, 2H), 7.20 (t, *J*= 8.9 Hz, 2H), 7.05 (ddd, *J*=9.0, 4.3, 1.5 Hz, 2H), 6.24 (s, 1H), 4.54–4.44 (m, 1H), 4.12 (d, *J*=19.7 Hz, 1H), 4.03 (d, *J*=5.0 Hz, 2H), 3.96 (d, *J*=16.6 Hz, 1H), 3.84 (d, *J*=12.4 Hz, 1H), 3.79–3.67 (m, 0H), 3.52 (s, 1H), 3.40–3.32 (m, 1H), 2.94 (s, 1H), 2.86 (d, *J* = 20.6 Hz, 1H).
^{13}C NMR (101 MHz, DMSO-d_6) δ 155.11, 133.70, 133.39, 127.44, 127.36, 116.72, 116.49, 116.44, 116.36, 116.25, 115.95, 115.73, 71.17, 64.52, 64.30, 57.86, 57.69, 51.80, 49.99, 49.93, 48.42, 45.94, 40.63, 40.42, 40.20, 39.99, 39.78, 39.56, 39.35, 24.06, 23.76.
^{19}F NMR: (565 MHz, DMSO-d_6) δ -114.37, -123.53 (2F).

Ro-A10; 4-[3-[4-(4-fluorophenyl)-3,6-dihydro-1(2H)-pyridinyl]-2-hydroxypropoxy]-phenyl

Ro-A10 was synthesized on a 0.75 mmol scale using **BB09**, and **PTP-F**. The synthesis was performed as described in **Ro 8-4304**. However, the reaction was stirred at reflux O/N. The next day, this was slowly cooled down to RT, before

placing it in the freezer O/N. The precipitate was filtered, and washed with EtOH. The obtained product was purified by column chromatography (eluent: DCM/MeOH 9/1). The obtained product was treated with 3 ml ethanolic HCl (1M), and this was stirred O/N. The ethanolic liquid was removed in vacuo, leaving a colorless solid, in a yield of 161 mg (66%).
HRMS *m/z* (ESI) 328.180, consistent with empirical formula $C_{20}H_{22}FNO_2$ with an accuracy of 0.0165 ppm (accepted as $[M+H]^+$).
^{1}H NMR (400 MHz, DMSO-d_6) δ 10.27 (s, 1H), 10.19 (s, 0H), 7.65–7.57 (m, 2H), 7.42–7.30 (m, 2H), 7.34–7.24 (m, 2H), 7.02 (ddd, *J*=8.4, 6.9, 2.6 Hz, 3H), 6.24 (s, 1H), 4.49 (s, 1H), 4.25–4.05 (m, 1H), 4.08–4.01 (m, 2H), 3.97 (d, *J*=17.2 Hz, 1H), 3.89–3.68 (m, 1H), 3.62–3.39 (m, 1H), 2.94 (s, 1H), 2.88–2.81 (m, 1H).
^{13}C NMR (101 MHz, DMSO-d_6) δ 158.71, 130.04, 127.45, 127.36, 121.43, 116.72, 116.44, 115.97, 115.75, 115.03, 70.41, 64.53, 64.29, 57.88, 57.66, 56.49, 51.85, 50.06, 49.87, 48.34, 24.07, 23.76.
^{19}F NMR: (565 MHz, DMSO-d_6) δ -114.32 (1F).

Ro-A11; 4-[3-[4-(4-fluorophenyl)-3,6-dihydro-1(2H)-pyridinyl]-2-hydroxypropoxy]-phenyl ethanone
Ro-A11 was synthesized on a 1 mmol scale using **BB10** and **PTP-F**. The synthesis was performed as described in **Ro 8-4304**. However, 2.5 Eq of TEA was used, and the reaction was stirred at reflux O/N. The next day, this was slowly cooled down to RT. An orange solution was visible. The EtOH was removed in vacuo, and the product was purified by column chromatography (eluent: EtOAc/ MeOH, 9/1), yielding 242 mg (66%) of product (beige solid).
HRMS *m/z* (ESI) 370.202, consistent with empirical formula $C_{22}H_{24}NO_3$ with an accuracy of 0.028 ppm (accepted as $[M+H]^+$).
^{1}H NMR (600 MHz, DMSO-d_6) δ 7.95–7.90 (m, 2H), 7.50–7.43 (m, 2H), 7.19–7.12 (m, 2H), 7.09–7.03 (m, 2H), 6.12 (dq, *J*=3.5, 1.8 Hz, 1H), 4.11 (dd, *J*=9.8, 3.5 Hz, 1H), 4.08–4.03 (m, 1H), 4.05–3.96 (m, 1H), 3.31 (s, 1H), 3.17 (q, *J*=3.1 Hz, 2H), 2.75 (dt, *J*=11.2, 5.6 Hz, 1H), 2.68 (dt, *J*=11.3, 5.7 Hz, 1H), 2.59 (dd, *J*=12.6, 6.1 Hz, 1H), 2.52 (s, 3H), 2.46 (dt, *J*=6.1, 3.3 Hz, 2H).
^{13}C NMR (151 MHz, DMSO-d_6) δ 196.74, 163.15, 162.59, 160.98, 137.09, 137.07, 133.39, 130.95, 130.27, 126.91, 126.86, 122.61, 115.59, 115.45, 114.80, 113.92, 71.82, 67.09, 66.99, 61.11, 53.87, 51.02, 27.95, 26.87.
^{19}F NMR: (565 MHz, DMSO-d_6) δ -115.93 (1F).

Ro-A12; 4-[3-[4-(4-fluorophenyl)-3,6-dihydro-1(2H)-pyridinyl]-2-hydroxypropoxy]-ethylphenoxy
Ro-A12 was synthesized on a 1 mmol scale using **BB11**, and **PTP-F**. The synthesis was performed as described in **Ro 8-4304**. However, the reaction was stirred at reflux O/N. The next day, this was slowly cooled down to RT. A precipitate was visible. This was filtered, and washed with a small amount of EtOH. The obtained product was treated with 3 ml ethanolic HCl (1M), and this was stirred for 1 h before removal in vacuo. This left a beige solid, in a yield of 52 mg (38%). This was used without any further purification.
HRMS *m/z* (ESI) 356.196, consistent with empirical formula $C_{22}H_{16}FNO_2$ with an accuracy of 0.0012 ppm (accepted as $[M+H]^+$).
^{1}H NMR (600 MHz, DMSO-d_6) δ 10.22 (s, 0H), 10.13 (s, 1H), 7.59–7.52 (m, 2H), 7.27–7.20 (m, 2H), 7.14 (d, *J*=8.1 Hz, 2H), 6.91–6.86 (m, 2H), 6.19 (s, 1H), 6.00 (s, 1H), 5.95 (s, 0H), 4.41 (s, 1H), 4.14–4.01 (m, 1H), 4.00–3.86 (m, 3H), 3.78 (d, *J*=12.1 Hz, 1H), 3.69 (d, *J*=12.2 Hz, 1H), 3.50–3.34 (m, 1H), 3.30 (s, 1H), 2.94–2.86 (m, 1H), 2.76 (t, *J*=15.9 Hz, 1H), 2.54 (q, *J*=7.6 Hz, 2H), 1.15 (t, *J*=7.6 Hz, 3H).

^{13}C NMR (151 MHz, DMSO-d_6) δ 161.54, 156.79, 136.67, 135.22, 133.70, 129.17, 127.43, 127.37, 116.73, 116.45, 115.92, 115.78, 114.92, 70.55, 64.53, 64.29, 57.90, 57.68, 51.85, 50.04, 49.85, 48.32, 27.92, 27.75, 24.07, 23.74, 16.42.
^{19}F NMR: (565 MHz, DMSO-d_6) δ -114.3 (1F).

Ro-A13; 4-[3-[4-(4-fluorophenyl)-3,6-dihydro-1(2H)-pyridinyl]-ethoxy]-benzamide
Ro-A13 was synthesized on a 1 mmol scale using **BB12**, and **PTP-F**. The synthesis was performed as described in **Ro 8-4304**. However, 2.5 Eq of TEA was used, and the reaction was stirred at reflux O/N. The next day, this was slowly cooled down to RT. The EtOH was removed in vacuo leaving an orange solution, this was treated with 3 ml ethanolic HCl (1M), and stirred for 1 h before removal in vacuo. The resulting product was purified by column chromatography (eluent: DCM/MeOH 9/1), yielding 211 mg (62%) of product (light yellow solid).
HRMS *m/z* (ESI) 341.167, consistent with empirical formula $C_{20}H_{21}FN_2O_2$ with an accuracy of 0.0083 ppm (accepted as $[M+H]^+$).
^1H NMR (600 MHz, DMSO-d_6) δ 9.79 (s, 2H), 7.88–7.83 (m, 2H), 7.56–7.50 (m, 2H), 7.25–7.19 (m, 2H), 7.02 (d, *J*=8.5 Hz, 2H), 6.17 (dq, *J*=3.5, 1.8 Hz, 1H), 3.74 (q, *J*=2.7 Hz, 2H), 3.30 (t, *J* = 6.0 Hz, 2H), 3.09 (s, 2H), 2.67 (tq, *J*=3.9, 2.0 Hz, 2H), 1.30 (s, 1H), 1.29 (ddd, *J*=12.8, 3.4, 0.0 Hz, 1H).
^{13}C NMR (151 MHz, DMSO-d_6) δ 167.82, 133.78, 133.47, 129.83, 127.33, 127.27, 117.39, 115.91, 115.77, 115.65, 115.50, 114.40, 45.92, 41.88, 40.51,31.97, 23.67, 15.67, 8.98.
^{19}F NMR: (565 MHz, DMSO-d_6) δ -114.54 (1F).

Ro-A14; 4-[3-[4-(4-fluorophenyl)-3,6-dihydro-1(2H)-pyridinyl]-propoxy]-benzamide
Ro-A14 was synthesized on a 1 mmol scale using **BB13**, and **PTP-F**. The synthesis was performed as described in **Ro 8-4304**. However, 2.5 Eq of TEA was used, and the reaction was stirred at reflux O/N. The next day, this was slowly cooled down to RT. A beige precipitate was visible, in an orange solution. This was filtered, and washed with EtOH. The obtained product was treated with 3 ml ethanolic HCl (1M), and this was stirred for 1 h before removal in vacuo. This left a slightly yellow solid, in a yield of 133 mg (38%). This was used without any further purification.
HRMS *m/z* (ESI) 355.189, consistent with empirical formula $C_{21}H_{23}FN_2O_2$ with an accuracy of 0.0146 ppm (accepted as $[M+H]^+$).
^1H NMR (400 MHz, DMSO-d_6) δ 10.74 (s, 1H), 7.91–7.81 (m, 2H), 7.61–7.51 (m, 2H), 7.28–7.18 (m, 2H), 7.19 (s, 1H), 7.04–6.96 (m, 2H), 6.19 (d, *J*=3.2 Hz, 1H), 4.16 (t, *J*=6.1 Hz, 2H), 4.04 (d, *J*=17.1 Hz, 1H), 3.87–3.59 (m, 0H), 3.70 (d, *J*=11.6 Hz, 1H), 3.39–3.30 (m, 2H), 3.25 (dd, *J*=14.3, 7.3 Hz, 1H), 2.96–2.84 (m, 1H), 2.75 (d, *J*=17.6 Hz, 1H), 2.27 (dd, *J*=10.7, 6.0 Hz, 2H, H-5).
^{13}C NMR (101 MHz, DMSO-d_6) δ 167.81, 161.14, 161.04, 135.21, 135.17, 133.64, 129.85, 127.45, 127.37, 127.23, 116.75, 115.96, 115.74, 114.37, 65.60, 52.78, 50.10, 48.57, 24.22, 23.96, 21.81.
^{19}F NMR: (565 MHz, DMSO-d_6) δ -114.34 (1F).

Ro-A15; 4-[3-[4-(4-fluorophenyl)-3,6-dihydro-1(2H)-pyridinyl]-butoxy]-benzamide
Ro-A15 was synthesized on a 1 mmol scale using **BB14**, and **PTP-F**. The synthesis was performed as described in **Ro 8-4304**. However, 2.5 Eq of TEA was used, and the reaction was stirred at reflux O/N. The next day, this was slowly cooled down to RT. A yellow precipitate was visible. This was filtered, and washed with EtOH. The obtained product was treated with 3 ml ethanolic HCl (1M), and this was stirred for 1 h before removal in vacuo. This left a beige solid, in a yield of 207 mg (61%). This was used without any further purification.

HRMS *m/z* (ESI) 369.207, consistent with empirical formula $C_{22}H_{25}FN_2O_2$ with an accuracy of 0.017 ppm (accepted as $[M+H]^+$).
^{1}H NMR (600 MHz, DMSO-d_6) δ 10.52 (s, 1H), 7.88–7.85 (m, 2H), 7.57–7.52 (m, 2H), 7.26–7.20 (m, 2H), 7.19 (s, 1H), 7.02–6.94 (m, 2H), 6.17 (p, *J*=2.2 Hz, 1H), 4.07 (dt, *J*=11.2, 6.1 Hz, 2H), 4.00 (d, *J*=18.9 Hz, 1H), 3.80–3.69 (m, 1H), 3.65 (dd, *J*=10.7, 5.0 Hz, 1H), 3.24 (ddqd, *J*=15.8, 9.4, 5.8, 3.8, 3.0 Hz, 3H), 2.90–2.81 (m, 1H), 2.74 (d, *J*=17.5 Hz, 1H), 1.97–1.76 (m, 4H).
^{13}C NMR (151 MHz, DMSO-d_6) δ 167.82, 161.53, 161.28, 135.22, 135.19, 133.63, 129.83, 127.42, 127.37, 127.01, 116.75, 115.92, 115.78, 114.34, 114.29, 67.47, 54.93, 50.03, 49.94, 48.49, 26.27, 24.16, 20.85, 8.94.
^{19}F NMR: (565 MHz, DMSO-d_6) δ -114.36 (1F).

Ro-A16; 4-[3-[4-(4-fluorophenyl)-3,6-dihydro-1(2H)-pyridinyl]-pentoxy]-benzamide
Ro-A16 was synthesized on a 1 mmol scale using **BB15**, and **PTP-F**. The synthesis was performed as described in **Ro 8-4304**. However, 2.5 Eq of TEA was used, and the reaction was stirred at reflux O/N. The next day, this was slowly cooled down to RT. The EtOH was removed in vacuo leaving an orange solution, this was treated with 3 ml ethanolic HCl (1M), and stirred for 1 h, before removal in vacuo. The resulting product was purified by column chromatography (eluent: DCM/MeOH 9/1), yielding 115 mg (30%) of product (yellow solid).
HRMS *m/z* (ESI) 383.214, consistent with empirical formula $C_{23}H_{27}FN_2O_2$ with an accuracy of 0.0083 ppm (accepted as $[M+H]^+$).
^{1}H NMR (600 MHz, DMSO-d_6) δ 7.87–7.82 (m, 2H), 7.83 (s, 1H), 7.58–7.53 (m, 2H), 7.26–7.20 (m, 2H), 7.00–6.95 (m, 2H), 6.18 (s, 1H), 4.06 (t, *J*=6.3 Hz, 2H), 4.00 (d, *J*=16.9 Hz, 1H), 3.80–3.73 (m, 1H), 3.66 (d, *J*=12.1 Hz, 1H), 3.23 (d, *J*=11.2 Hz, 2H), 3.17 (s, 3H), 2.82 (s, 1H), 1.79 (s, 3H), 1.49 (p, *J*=7.7 Hz, 2H).
^{13}C NMR (151 MHz, DMSO-d_6) δ 167.83, 133.67, 129.83, 127.45, 127.39, 116.78, 115.93, 115.79, 114.28, 67.74, 55.41, 55.14, 46.00, 23.50, 23.14, 8.99.
^{19}F NMR: (565 MHz, DMSO-d_6) δ -114.35 (1F).

Ro-A17; 4-[3-[4-(4-fluorophenyl)-3,6-dihydro-1(2H)-pyridinyl]-2-hydroxybutoxy]-benzamide
RO-A17 was synthesized on a 1 mmol scale using **BB16**, and **PTP-F**. The synthesis was performed as described in **Ro 8-4304**. However, the reaction was stirred at reflux O/N. The next day, this was slowly cooled down to RT. An orange solution was visible. EtOH was removed in vacuo, and the obtained product was purified by column chromatography (eluent: EtOAc/MeOH 9/1). However, the product was stuck on the column, thus the eluent was changed to 100% MeOH. This gave a yellow solid, in a yield of 29 mg (9%). This was still contaminated with **PTP-F**, however since the yield was so low, no further purification was done. Probably a revere phase column purification would have been better.
HRMS *m/z* (ESI) 385.192, consistent with empirical formula $C_{22}H_{25}FN_2O_3$ with an accuracy of 0.0071 ppm (accepted as $[M+H]^+$).
^{1}H NMR (600 MHz, DMSO-d_6) δ 7.93–7.87 (m, 3H), 7.61–7.55 (m, 2H), 7.31–7.25 (m, 2H), 7.07–7.02 (m, 2H), 6.23 (td, *J*=3.6, 1.8 Hz, 1H), 4.19 (q, *J*=5.2 Hz, 1H), 4.05–3.94 (m, 4H,; 3.76 (q, *J*=2.7 Hz, 2H), 3.32 (t, *J*=6.0 Hz, 2H), 3.23 (d, *J*=5.1 Hz, 2H), 2.80 (s, 1H), 2.73 (tt, *J*=6.2, 3.0 Hz, 2H).
^{13}C NMR (151 MHz, DMSO-d_6)) δ 167.87, 161.49, 133.47, 129.81, 127.29, 127.24, 126.99, 126.94, 115.88, 115.74, 115.63, 115.49, 114.38, 113.92, 72.67, 67.60, 41.84, 40.51, 40.48, 23.76, 17.72.
^{19}F NMR: (565 MHz, DMSO-d_6) δ -114.62 (1F).

Ro-A18; 4-[3-[4-(4-fluorophenyl)-3,6-dihydro-1(2H)-pyridinyl]-2-hydroxy-2-methyl-propoxy]-benzamide

RO-A18 was synthesized on a 1 mmol scale using **BB17**, and **PTP-F**. The synthesis was performed as described in **Ro 8-4304**. However, the reaction was stirred at reflux O/N. The next day, this was slowly cooled down to RT. A precipitate was visible. This was filtered, and washed with a small amount of EtOH. The obtained beige product was purified by column chromatography (eluent: EtOAc/MeOH 9/1). This left a beige solid, in a yield of 39 mg (10%). This was used without any further purification.

HRMS *m/z* (ESI) 385.196, consistent with empirical formula $C_{22}H_{25}FN_2O_3$ with an accuracy of 0.0111 ppm (accepted as $[M+H]^+$).

^{1}H NMR (600 MHz, DMSO-d_6) δ 7.92–7.86 (m, 2H), 7.87 (s, 1H), 7.54–7.47 (m, 2H), 7.21 (d, *J*=4.0 Hz, 1H), 7.21–7.16 (m, 2H), 7.06–7.03 (m, 2H), 6.17–6.13 (m, 1H); 4.12–4.06 (m, 1H), 4.00 (dd, *J*=18.8, 9.2 Hz, 2H), 3.87 (d, *J*=9.0 Hz, 1H), 3.33–3.23 (m, 2H), 2.85 (dt, *J*=11.3, 5.6 Hz, 1H), 2.79 (dt, *J*=11.3, 5.6 Hz, 1H), 2.45 (s, 2H), 1.27 (s, 3H).

^{13}C NMR (151 MHz, DMSO- d_6) δ 167.88, 167.82, 162.54, 161.66, 161.50, 160.93, 137.08, 137.06, 133.22, 129.83, 129.79, 127.06, 126.94, 126.90, 126.84, 126.78, 123.16, 115.55, 115.41, 114.44, 114.44, 114.03, 113.92, 73.89, 73.67, 72.59, 72.31, 70.79, 64.45, 58.39, 55.20, 52.17, 40.52, 29.10, 28.08, 23.98, 22.60.

^{19}F NMR: (565 MHz, DMSO-d_6) δ -116.01 (1F).

References

(1) Edenhofer, A.; Spiegelberg, H. [4'-(Phenyl-3,6-Dihydro-1-(2H)Pyridyl]-2-Hydroxypropoxy-Anilides and Derivatives Thereof. United State Patents no. 3,674,799A, 1972.

(2) Conway, R. J.; Valant, C.; Christopoulos, A.; Robertson, A. D.; Capuano, B.; Crosby, I. T. Synthesis and SAR Study of 4-Arylpiperidines and 4-Aryl-1,2,3,6-Tetrahydropyridines as 5-HT2C Agonists. *Bioorg. Med. Chem. Lett.* **2012**, *22*, 2560–2564.

(3) Junker, A.; Balasubramanian, R.; Ciancetta, A.; Uliassi, E.; Kiselev, E.; Martiriggiano, C.; Trujillo, K.; Mtchedlidze, G.; Birdwell, L.; Brown, K. A.; et al. Structure-Based Design of 3-(4-Aryl-1H-1,2,3-Triazol-1-Yl)-Biphenyl Derivatives as P2Y14 Receptor Antagonists. *J. Med. Chem.* **2016**, *59*, 6149–6168.

(4) Gessner, W.; Brossi, A.; Shen, R.; Abell, C. W. Synthesis and Dihydropteridine Reductase Inhibitory Effects of Potential Metabolites of the Neurotoxin 1-Methyl-4-Phenyl-1,2,3,6-Tetrahydropyridine. *J. Med. Chem.* **1985**, *28* (3), 311–317.

(5) Arnáiz, F. J. A Convenient Way to Generate Hydrogen Chloride in the Freshman Lab. *J. Chem. Educ.* **1995**, *72* (12), 1139.

(6) Kubota, H.; Kakefuda, A.; Watanabe, T.; Ishii, N.; Wada, K.; Masuda, N.; Sakamoto, S.; Tsukamoto, S. Synthesis and Pharmacological Evaluation of 1-Oxo-2-(3-Piperidyl)-1,2,3,4- Tetrahydroisoquinolines and Related Analogues as a New Class of Specific Bradycardic Agents Possessing If Channel Inhibitory Activity. *J. Med. Chem.* **2003**, *46*, 4728–4740.

(7) Ray, N. C.; Bull, R. J.; Finch, H.; Heuvel, M. Van Den; Bravo, J. A. Oxazole and Thiazole Derivatives and Their Uses. WO 2008/096093 A1, 2008.

Supporting Table 1. Primers used for PCR experiments

ID	Gene	Sequence (5' to 3')
AT5G60390	EF1α_F	CTCTTCTTGCTTTCACCCTTGG
	EF1α_R	ACCTAGCCTTGGAGTATTTGGG
AT1G13440	GADPH_F	GACTGGAGAGGTGGAAGAGC
	GADPH_R	GGCAACACTTTCCCAACAGC
AT2G14610	PR1_F	CAACTACGCTGCGAACACG
	PR1_R	GGCACATCCGAGTCTCACTG
AT1G02450	NIMIN1_F	AGGAGGAAATCTAACGGCGG
	NIMIN1_R	AACCCGTACGACACTGAGAG
AT3G48090	EDS1_F	CGTTCAAGCTGCATTAGAGGAAG
	EDS1_R	ACCTCTCTTGCTCGATCAC
AT3G52430	PAD4_F	CGGCTACCAACAACAACCAC
	PAD4_R	CATTCCCGGAGGTAAGTTCG
AT3G57260	PR2_F	GCTTCCTTCTTCAACCACACAG
	PR2_R	TGGACTTGGCAAGGTATCGC
AT1G74710	Sid2_F	TGGCAAGATCGCTGTTGAAT
	Sid2_R	AGCCAACATTGAACTTCCACC
AT5G46210	Cul4_F	GCTGGCTGTTTCCCTGTTTC
	Cul4_R	CAGCTCCTTGTCCTCTATGC
AT2G28390	Sand_F	AACTCTATGCAGCATTTGATCCAC
	Sand_R	TGATTGCATATCTTTATCGCCATC

Supporting Table 2. Starting compounds for synthesis of Ro 8-4304 derivatives

Derivative	Original starting compound	was changed for:
Ro-A01	PTP-F	(4-(4-bromophenyl)-1,2,3,6-tetrahydropyridine)
Ro-A02	PTP-F	(4-(4-hydroxy-phenyl)-1,2,3,6-tetrahydropyridine)
Ro-A03	PTP-F	4-phenyl-1,2,3,6-tetrahydropyridine
Ro-A04	PTP-F	1,2,3,6-tetrahydropyridine
Ro-A05	PTP-F	4-[4-(trifluoromethyl)phenyl]4-piperidinol
Ro-A06	PTP-F	4-[4-(trifluoromethyl)-phenyl]-1,2,3,6-tetrahydropyridine
Ro-A07	4-hydroxybenzamide	tert-Butyl(4-hydroxybenzyl)carbamate
Ro-A08	4-hydroxybenzamide	tert-Butyl(4-hydroxybenzyl)carbamate
Ro-A09	4-hydroxybenzamide	4-fluorophenol
Ro-A10	4-hydroxybenzamide	Phenol
Ro-A11	4-hydroxybenzamide	4-hydroxyacetophenon
Ro-A12	4-hydroxybenzamide	4-ethylphenol
Ro-A13	Epichlorohydrine	1,2-dibromoethane
Ro-A14	Epichlorohydrine	1,3-dibromopropane
Ro-A15	Epichlorohydrine	1,4-dibromobutane
Ro-A16	Epichlorohydrine	1,5-dibromopentane
Ro-A17	Epichlorohydrine	2-(2-chloroethyl)oxirane
Ro-A18	Epichlorohydrine	2-(chloromethyl)-2-methyloxirane

Figure S1

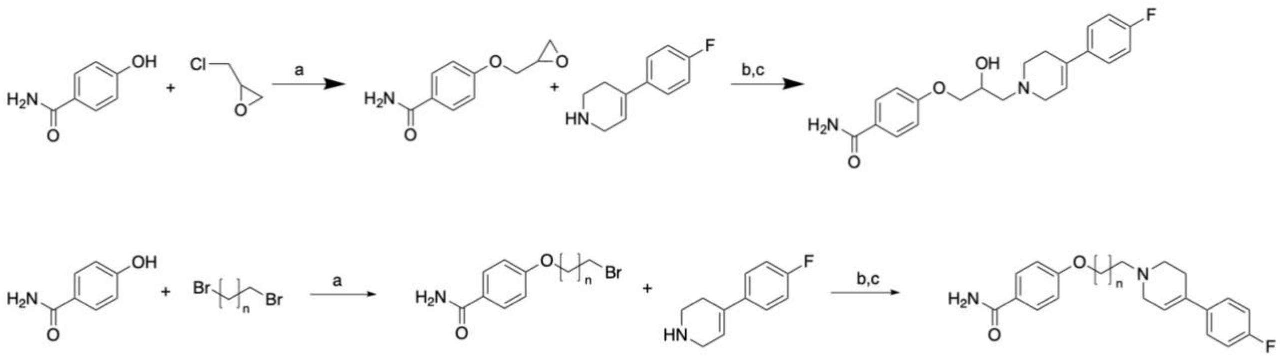

Figure S1. Synthesis of derivatives of Ro8-4304. Upper lane: the synthesis of Ro8-4304, and derivatives, as described (Edenhofer, A.; Spiegelberg, H. [4'-(Phenyl-3,6-Dihydro-1-(2H)Pyridyl]-2-Hydroxypropoxy-Anilides and Derivatives Thereof. United State Patents no. 3,674,799A, 1972). Reagents and conditions: (a) 4-hydroxybenzamide and epichlorohydrine, or similar compounds, were reacted overnight at room temperature, in a solution of NaOH. (b) The synthesized 4-oxiranylmethoxy benzamide was combined with e.g. 4-(4-fluorophenyl)-1,2,3,6-tetrahydropyridine·HCl in EtOH, pre-treated with triethylamine. The mixture was heated to reflux for 2 h, and Ro 8-4304 precipitated upon cooling. (c) Ro 8-4304 was treated with 1M ethanolic HCl, to form its hydrochloride salt. Lower lane: the adapted synthesis of Ro-A13 to Ro-A16 (Kubota et al., *J. Med. Chem.* 2003, *46*, 4728–4740 and Ray, N. C.; Bull, R. J.; Finch, H.; Heuvel, M. Van Den; Bravo, J. A. Oxazole and Thiazole Derivatives and Their Uses. WO 2008/096093 A1, 2008). Reagents and conditions: (a) 4-hydroxybenzamide and eg. 1,3-dibromopropane were reacted overnight at reflux, in the presence of K_2CO_3, in ACN. (b) The product, 4-(4-bromopropoxy)benzamide, and 4-(4-fluorophenyl)-1,2,3,6-tetrahydropyridine·HCl were combined in EtOH, pre-treated with triethylamine. The mixture was heated to reflux overnight, and the derivative precipitated upon cooling. (c) The derivative was treated with 1M ethanolic HCl, to form its hydrochloride salt.

Figure S2

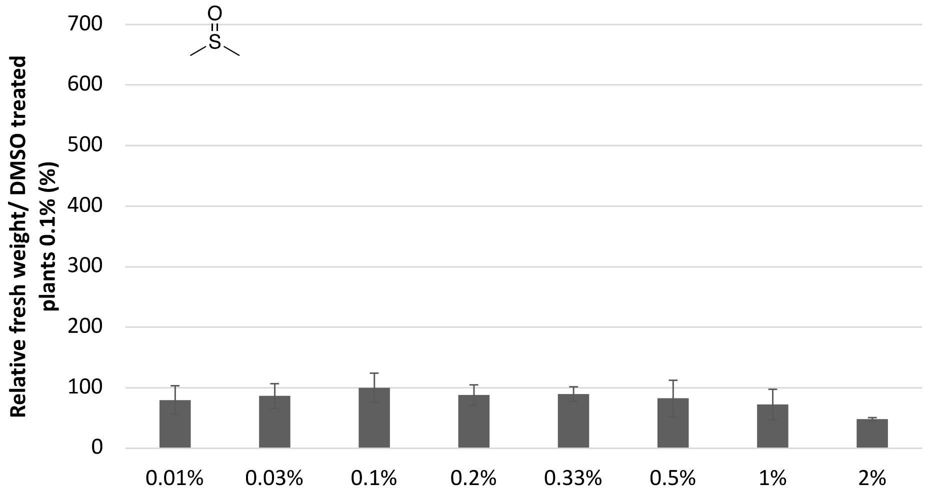

O
S
Relative fresh weight/ DMSO treated plants 0.1% (%)
700
600
500
400
300
200
100
0
0.01%
0.03%
0.1%
0.2%
0.33%
0.5%
1%
2%

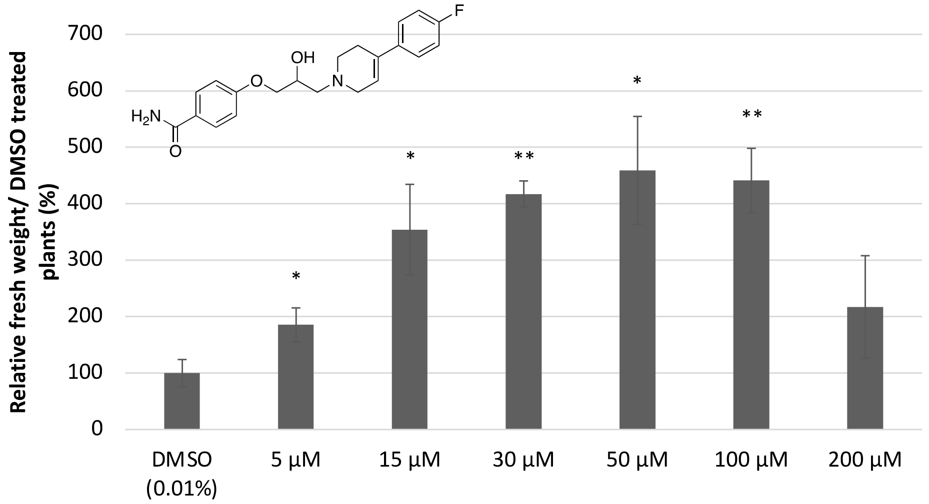

F
OH
O
N
H_2N
O
Relative fresh weight/ DMSO treated plants (%)
700
600
500
400
300
200
100
0
*
*
**
*
**
*
DMSO (0.01%)
5 µM
15 µM
30 µM
50 µM
100 µM
200 µM

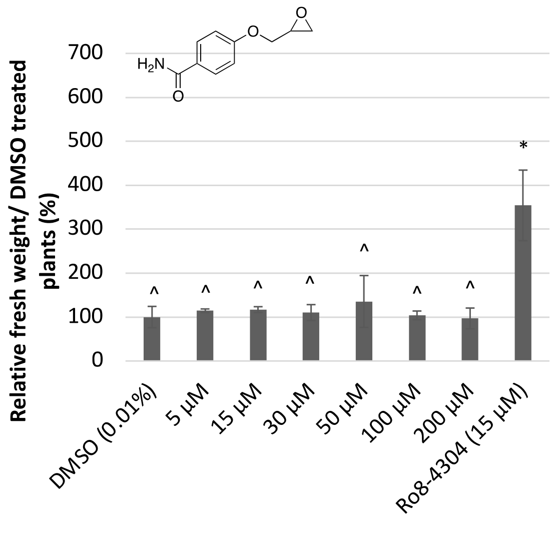

H_2N
O
O
O
Relative fresh weight/ DMSO treated plants (%)
700
600
500
400
300
200
100
0
^
^
^
^
^
^
^
*
DMSO (0.01%)
5 µM
15 µM
30 µM
50 µM
100 µM
200 µM
Ro8-4304 (15 µM)

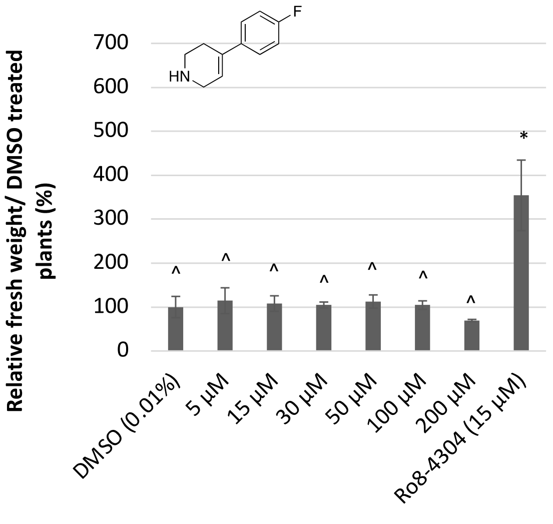

F
HN
Relative fresh weight/ DMSO treated plants (%)
700
600
500
400
300
200
100
0
^
^
^
^
^
^
^
*
DMSO (0.01%)
5 µM
15 µM
30 µM
50 µM
100 µM
200 µM
Ro8-4304 (15 µM)

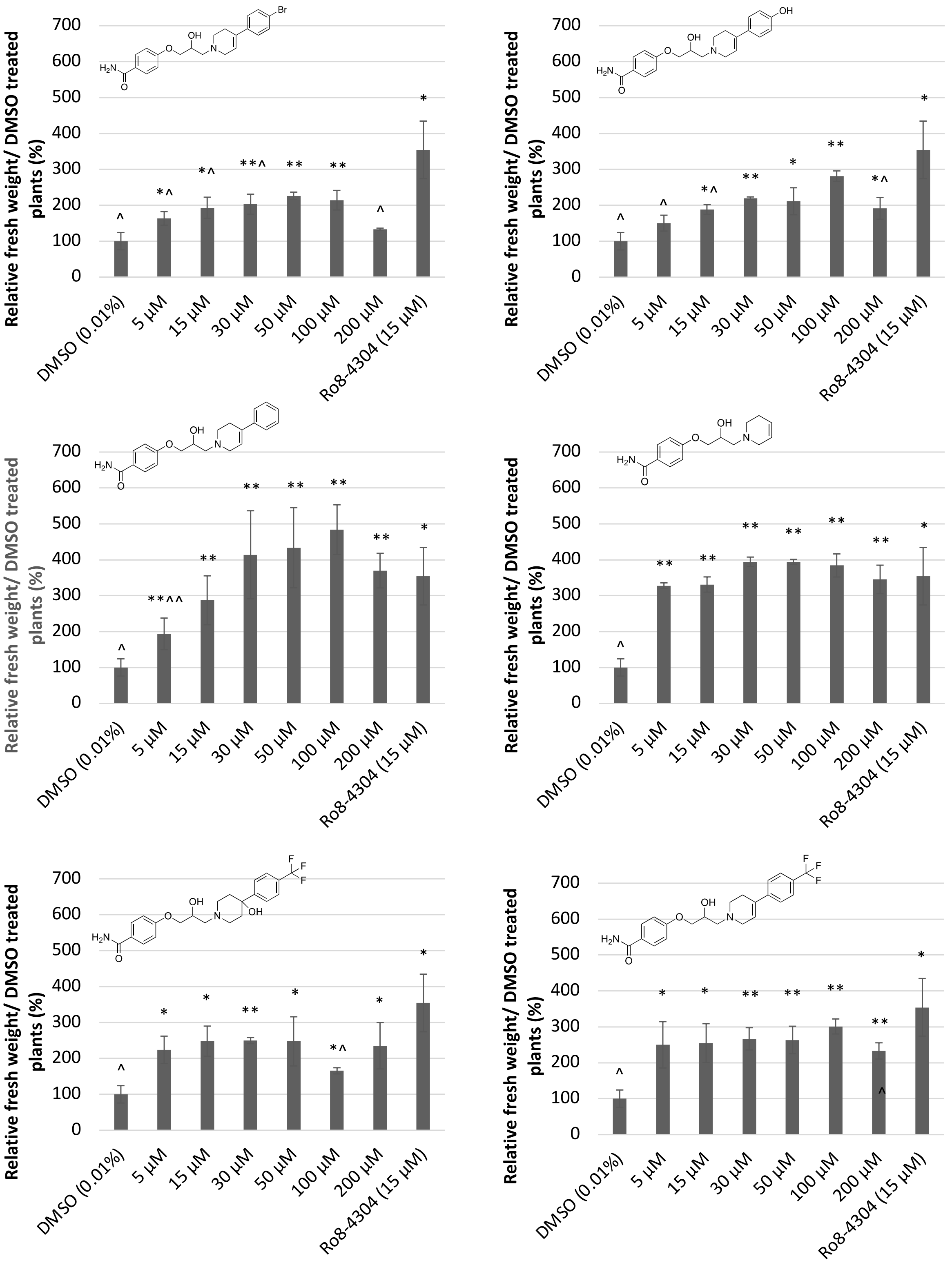
Relative fresh weight/ DMSO treated plants (%)
0
100
200
300
400
500
600
700
DMSO (0.01%)
5 µM
15 µM
30 µM
50 µM
100 µM
200 µM
Ro8-4304 (15 µM)
H2N
O
OH
N
Br
F
F
F

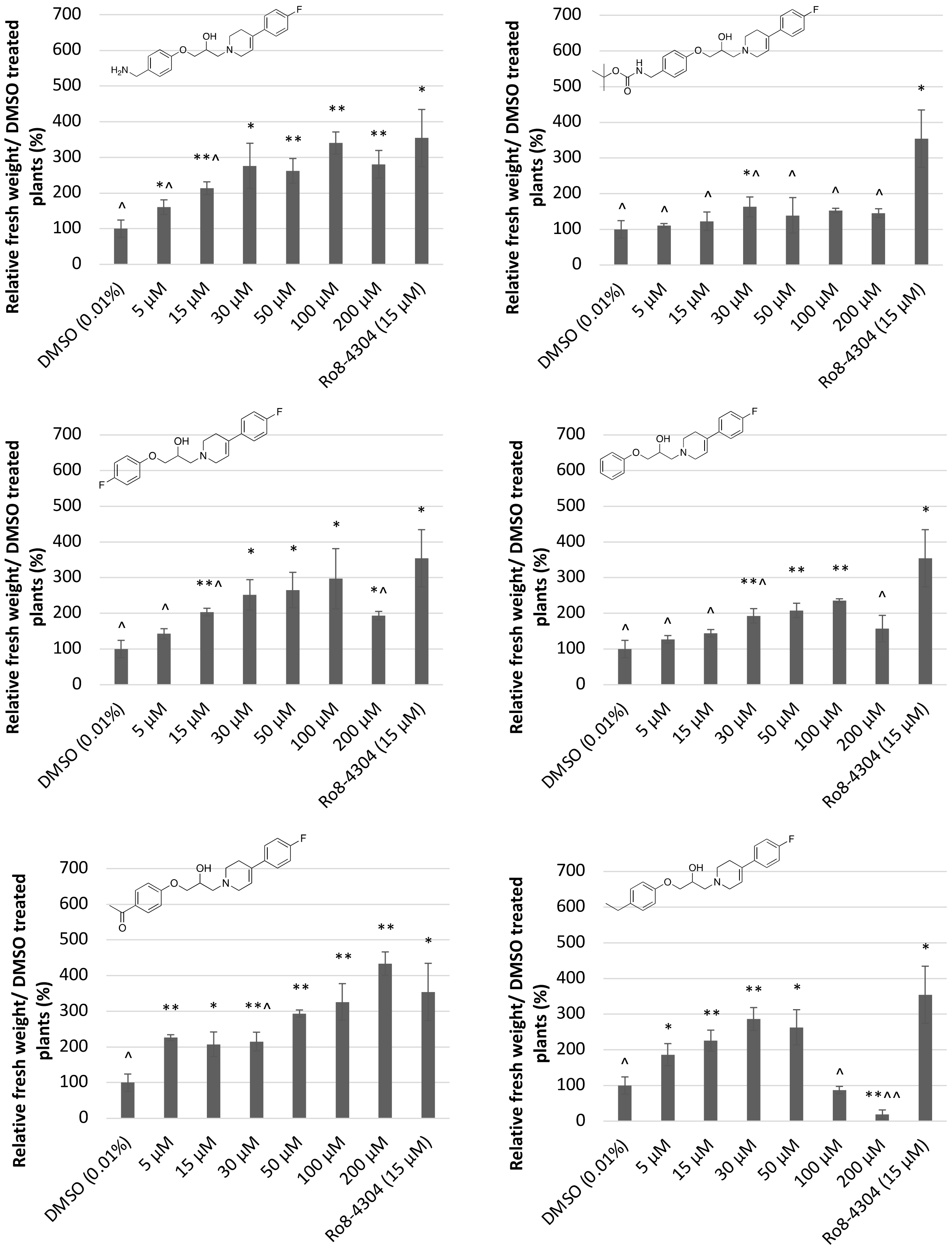
Relative fresh weight/ DMSO treated plants (%)
0
100
200
300
400
500
600
700
DMSO (0.01%)
5 µM
15 µM
30 µM
50 µM
100 µM
200 µM
Ro8-4304 (15 µM)
OH
N
O
F
H2N
O
NH

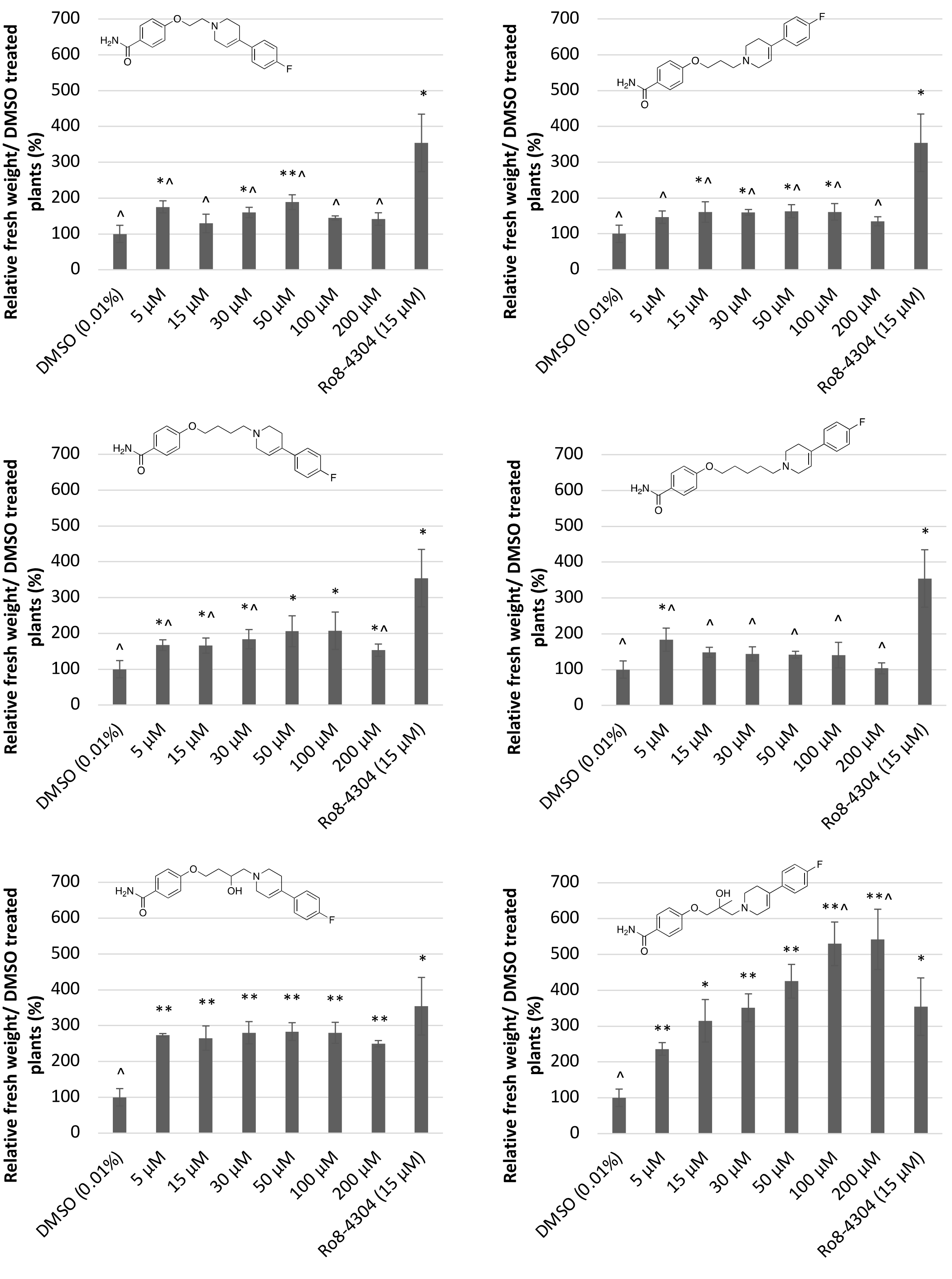

Figure S2. The relative fresh weight of *chs3-2D* treated with different concentrations of DMSO, Ro-8-4304, 4-oxiranyl methoxybenzamide, and PTP-F, as well as the derivatives R0-A01 to Ro-A18. Seedlings of *chs3-2D* were grown at 18°C for 21 days. Data represent mean ± SD (n = 3, with 8 to 12 plants each). One asterisk indicates a statistical difference of $p < 0.05$, while two asterisks indicate a statistical difference of $p < 0.01$, relative to the negative DMSO control. The caret symbol uses the same system relative the positive control; one caret indicates a difference at $p < 0.05$ and two carets indicate a difference at $p < 0.01$. The significances were calculated using the Student’s t-test.

Figure S3

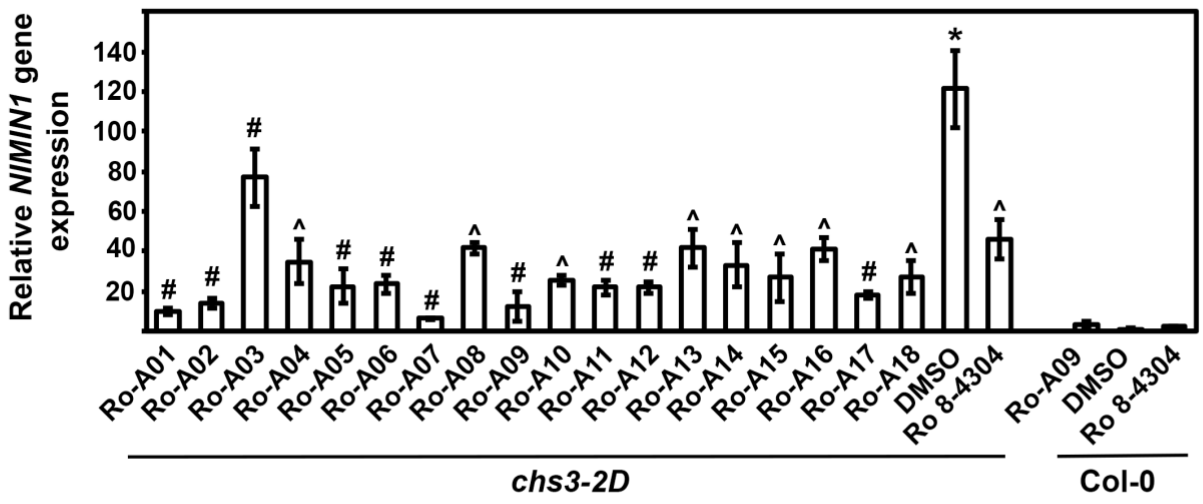

Figure S3. Relative expression of the *NIMIN1* gene in *chs3-2D* seedlings grown at 18°C for 21 days and treated with the respective compound at a concentration of 15 µM. Mean values of three replicates are given, and error bars represent the standard deviation. The carets indicate a significant difference of $p < 0.05$ in comparison to the values of *chs3-2D* treated with 0.01% DMSO. The asterisks indicate a significant difference of $p < 0.05$ in comparison to the values of *chs3-2D* treated with 15 µM Ro 8-4304. The hashtags indicate a significant difference of $p < 0.05$ in comparison to both, to the values of chs3-2D treated with 0.01% DMSO and to the values of chs3-2D treated with 15 µM Ro 8-4304.The significances were calculated using the Student's t-test.
